# A century and a half precipitation oxygen isoscape for China generated using data fusion and bias correction

**DOI:** 10.1038/s41597-023-02095-1

**Published:** 2023-04-06

**Authors:** Jiacheng Chen, Jie Chen, Xunchang J. Zhang, Peiyi Peng, Camille Risi

**Affiliations:** 1 State Key Laboratory of Water Resources & Hydropower Engineering Science, Wuhan University, Wuhan, 430072 China; 2 Hubei Key Laboratory of Water System Science for Sponge City Construction, Wuhan University, Wuhan, 430072 China; 3 USDA-ARS Oklahoma and Central Plains Agricultural Research Center, 7207W. Cheyenne St., El Reno, OK 73036 USA; 4 Chongqing Southwest Research Institute for Water Transport Engineering, Chongqing Jiaotong University, Chongqing, 400016 China; 5 Laboratoire de Meteorologie Dynamique, IPSL, CNRS, Ecole Normale Superieure, Sorbonne Universite, PSL Research University, Paris, France

**Keywords:** Hydrology, Hydrology, Stable isotope analysis

## Abstract

The precipitation oxygen isotopic composition is a useful environmental tracer for climatic and hydrological studies. However, accurate and high-resolution precipitation oxygen isoscapes are currently lacking in China. In this study, a precipitation oxygen isoscape in China for a period of 148 years is built by integrating observed and iGCMs-simulated isotope compositions using an optimal hybrid approach of three data fusion and two bias correction methods. The temporal and spatial resolutions of the isoscape are monthly and 50–60 km, respectively. Results show that the Convolutional Neural Networks (CNN) fusion method performs the best (correlation coefficient larger than 0.95 and root mean square error smaller than 1‰), and the other two data fusion methods perform slightly better than the bias correction methods. Thus, the isoscape is generated by using the CNN fusion method for the common 1969–2007 period and by using the bias correction methods for remaining years. The generated isoscape, which shows similar spatio-temporal distributions to observations, is reliable and useful for providing strong support for tracking atmospheric and hydrological processes.

## Background & Summary

Taking advantage of the fact that isotope composition varies sensitively with environmental conditions, environmental isotopes play an important role in the identification and characterization of the Earth’s systems processes^1^. The study of the hydrologic cycle is one of the most important applications of stable isotopes. Firstly, the isotope composition of water provides an effective tracking method of water sources. In the process of moisture transport, the isotope composition changes with atmospheric processes, which can reflect moisture contribution^2–4^. In addition, for surface runoff, soil water and groundwater, the isotope composition can also reflect the water source, infiltration mechanism and evaporation consumption of each system^5–8^. Secondly, isotope composition can also reveal hydrological processes that cannot be achieved by other methods^9^. For example, evaporation processes can be better diagnosed by dual hydrogen-oxygen or triple oxygen isotope, which can be used to quantify the raindrop re-evaporation^10,11^. Thirdly, isotopes can be incorporated into surface hydrology models as diagnostic tools. The isotope composition of evapotranspiration, soil moisture, and runoff can be predicted by incorporating the isotope cycle, thus the distribution of isotopic variation in evapotranspiration and runoff can be better understood^12^. What’s more, isotope composition can quantify evaporation rates, which is useful for understanding water balance and climate change from catchment to continental scales^1^. Precipitation isotopes can also be used to estimate the precipitation isotopic lapse rate by establishing relationships with climatic elements or elevation, so as to study paleoclimate and paleoelevation^13,14^.

The international observation of stable isotopes in precipitation began in the 1950s, and the Global Network of Isotopes in Precipitation (GNIP) established in 1961 provides first-hand data for the study of stable isotopes in precipitation. Since then, Austria (Austrian Network of Isotopes in Precipitation, ANIP)^15^, the United States (United State Network of Isotopes in Precipitation, USNIP)^16^, Switzerland (Swiss National Network for the Observation of Isotopes in the Water Cycle, NISOT)^17^, Canada (Canadian Network of Isotopes in Precipitation, CNIP)^18^ and other countries have also established their national networks, which provide strong data support for promoting and deepening the study of stable precipitation isotope.

The establishment of the isotope observation network in China was relatively late. Before 1985, GNIP had only one station in Hong Kong of China, and it was not until 1985 that more stations were selected for inclusion in GNIP. Due to the scarcity of stations on the Tibetan Plateau, the Chinese Academy of Sciences (CAS) launched the Tibetan Plateau Network of Isotopes in Precipitation (TNIP) in 1991^19^. However, most Chinese stations in GNIP stopped monitoring in the early 2000s^20^, and until 2004 only one station remained. In order to continue the systematic study, the CAS established the Chinese Network of Isotopes in Precipitation (CHNIP) based on the Chinese Ecosystem Research Network (CERN) in 2004^21^. Due to the difficulty and high cost of measuring precipitation isotope ratios^22^, most of the observed data are short in length. The spatial distribution of observation stations is uneven, with few stations in inaccessible areas^23^.

The stable isotopes in precipitation can also be simulated by isotope-equipped general circulation models (iGCMs). In contrast to observations, iGCMs can provide time-continuous and space-regular isotope data^24^. Joussaume, *et al*.^25^ incorporated the fractionation process of water stable isotope into GCM for the first time. They used the GCM of the Laboratoire de Météorologie Dynamique (LMD) to simulate the distribution of global water stable isotope, and the relationship between simulated precipitation oxygen isotope and meteorological elements was in good agreement with the measured results. Since then, an increasing number of GCMs have incorporated isotope cycles, for example, the ECHAM4 developed by the Max Planck Institute for Meteorology (MPI) in Germany^26^, the GISS E developed by the NASA Goddard Institute for Space Studies (GISS) in the United States^27,28^, the HadAM3 developed by the Hadley Centre for Climate Prediction and Research in the United Kingdom^29^, the LMDZ4 developed by the Laboratoire de Météorologie Dynamique in France^30^, and the MIROC32 developed by the Center for Climate System Research (CCSR) of the University of Tokyo in Japan^31^, etc.

On the basis of these, the comparison and evaluation of iGCMs in simulating isotopes have been conducted in many studies. Yoshimura, *et al*.^32^ indicated that due to the limitation of spatial and temporal resolution, iGCMs are poor in simulating the short-term (days) variability of stable isotopes in precipitation, while they are good at the monthly or annual scale. Conroy, *et al*.^33^ evaluated the spatio-temporal pattern of precipitation isotope variability in the tropical Pacific for iGCM simulations, and found that nudging models by reanalysis wind has a certain effect on precipitation isotope values, and the performance of models varies with regions. Zhang, *et al*.^34^ selected four iGCMs to evaluate the average precipitation isotopic composition in East Asia. The results showed that the characteristics of measured values were well reproduced by iGCM simulation, but the simulated values were all lower in the inland areas at middle and high latitudes, and the amount effect in arid areas was incorrectly simulated. Wang, *et al*.^23^ verified iGCM-simulated stable isotopes in precipitation in arid Central Asia. In general, the seasonality of stable isotopes in precipitation could be well simulated, but the values of oxygen isotopes were higher in summer and lower in winter, lower in the eastern section and higher in the western section. Che, *et al*.^35^ concluded that nudged simulation by LMDZ has the best comprehensive performance by comparing the simulated values of different models with the measured values of GNIP in China. In terms of altitude effects, CAM and GISS E perform better, while in terms of continental effects, the free simulations by GISS E and LMDZ perform better.

To comprehensively consider the error characteristics and advantages of different sources of data to reduce uncertainty, data fusion is usually used. One of the common methods for data fusion is to use *in-situ* observations as baselines to correct estimates from other sources. Several data fusion methods such as cokriging^36^, probability matching^37^, statistical objective analysis^38^, Bayesian correction^39^, probability density function–optimal interpolation^40^, and variational^41^ are usually used to fuse *in-situ* observation information. The key of these methods is to deal with the estimation errors directly based on weighted average, regression analysis, filtering analysis and other mathematical approaches. In contrast, neural network methods have stronger learning and generalization abilities, and have advantages in discovering complex relationships in data and processing large amounts of data^42^. So far, the neural network was mainly applied to precipitation data fusion in the field of hydrology but very little in isotopic hydrology. For example, Turlapaty, *et al*.^43^ used Artificial Neural Network to fuse various satellite precipitation products, and found the fusion performance was statistically superior to each individual dataset for all seasons. Sun and Tang^44^ combined information from satellite precipitation products and reanalysis data in Central Texas, U.S., by using an attention-based deep convolutional neural network (AU-Net), and found the Au-net models have achieved varying degrees of success under different climatic conditions. Wu, *et al*.^45^ combined Convolutional Neural Network with Long Short-Term Memory Network to fuse the TRMM satellite data, thermal infrared images of Gridded satellite, rain gauge data and elevation data. The results showed that this method can improve the accuracy of original TRMM data in China, even for regions with different precipitation intensities or sparse gauges.

Overall, both observations and iGCM simulations have advantages and disadvantages. The effort of constructing a database by taking advantages and circumventing disadvantages of both becomes a challenge. With the motivations of resolving the lack and uneven distribution of observations, as well as the coarse and biased iGCM simulations, this study aims to take a hybrid approach that makes full use of observations to integrate the advantages of various iGCMs by using the optimal combination of data fusion and bias correction methods. In order to determine the best scheme to build the dataset, two bias correction methods (BCMs) and three neural network data fusion methods (DFMs) are first compared in terms of bias correcting and fusing iGCM simulations. The new isoscape in monthly temporal and approximately 0.5° spatial resolutions is produced by combining the optimal data fusion and bias correction methods for the 1870–2017 period. The spatial and temporal distribution characteristics of oxygen isotopes in precipitation are then analysed for China.

## Methods

### Study area

**Fig. 1:**
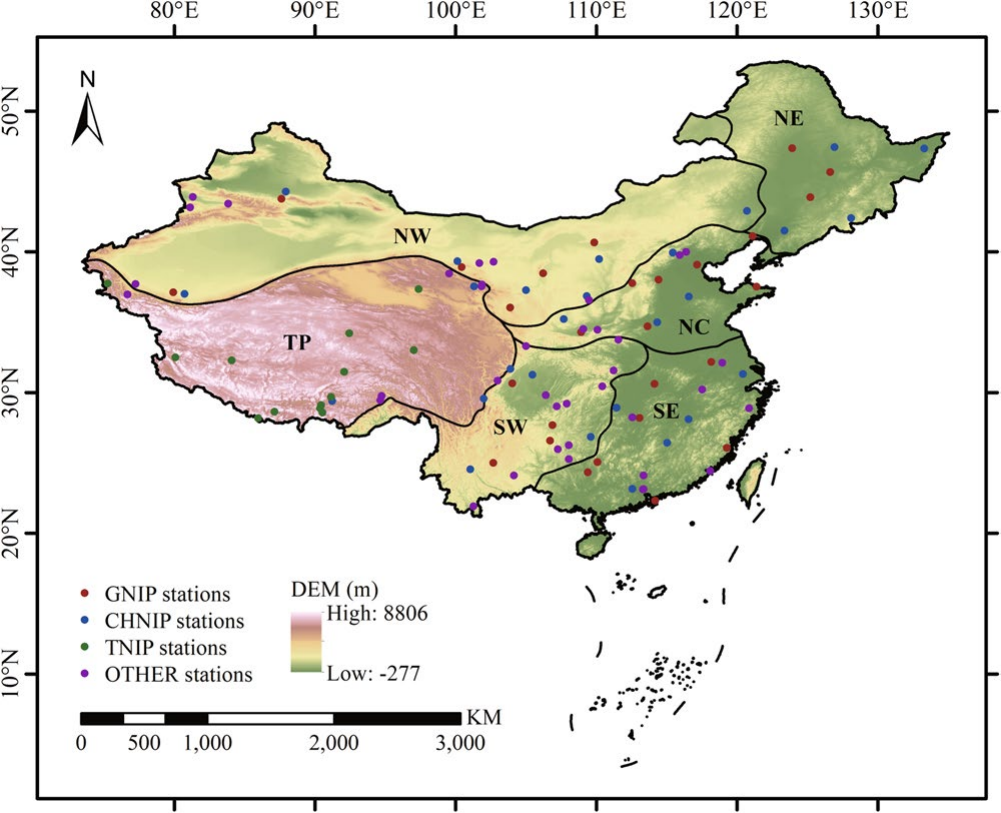
Map of the station locations and topography in the mainland of China. The dots indicate the distribution of isotope observation stations, with different colours representing different sources. The six sub-regions are plotted (NE – Northeast China, NC – North China, SE – Southeast China, SW – Southwest China, TP – Tibetan Plateau, NW – Northwest China).

China is located in the east of Eurasia and on the west coast of the Pacific Ocean. The topography of China generally presents three steps descending to the east. The climate is complex and diverse in China. Heavily influenced by the continents and oceans, the monsoon climate is significant, especially for the east of China. The spatiotemporal variation of precipitation stable isotopes is very complex due to the significant changes in winter and summer circulation^46^. The mainland of China can be geographically classified into three sub-regions, the eastern monsoon region, the arid northwest region and the Qinghai-Tibet Plateau region, according to topography, climate, soil and vegetation. The eastern monsoon region is further divided into four sub-regions by taking terrain and climatic conditions into account, as well as ensuring the sufficient number of stations and data volumes in a sub-region. To sum up, the study area is divided into six sub-regions for our analysis: Northeast China (NE), North China (NC), Southeast China (SE), Southwest China (SW), Qinghai-Tibet Plateau (TP) and Northwest China (NW), as shown in Fig. 1.

### Datasets

There are 107 oxygen isotope observation stations in the study area (Fig. 1), including 29 GNIP stations^47^ (available at https://nucleus.iaea.org/wiser), 27 CHNIP stations^46^, 13 TNIP stations^48,49^ and 38 stations from other sources (mainly from references). Monthly oxygen isotope composition of precipitation (δ^18^O_p_) is used for analysis. For a few references providing event isotope data, monthly precipitation weighted data are used. The time span of GNIP data mostly ranges from 1980 to 2000, and the length of the time period is basically 5–15 years. For CHNIP, most time periods are about 2–5 years ranging from 2005 to 2010. Most TNIP data are between 1995 and 2005, with varying lengths. More details about the observation stations can be found in Table S1.

Some physical-based ancillary data are introduced in the fusion methods, including elevation and meteorological data, to enrich the climate and terrain information in the process of data fusion. For regions with high spatial autocorrelation of isotope values, and regions with large variability in topography or climate, it is very necessary to introduce ancillary data^50,51^. Moreover, the neural network methods have the ability to integrate simulated data, observed data and ancillary data to extract enough spatial variability to generate more accurate oxygen isoscape^52^. The Digital Elevation Model (DEM) with a spatial resolution of 90 m is derived from the USGS/NASA Shuttle Radar Topographic Mission^53^ (SRTM, https://srtm.csi.cgiar.org/). The monthly temperature and precipitation gridded datasets on 0.5° spatial resolution in China developed by the National Meteorological Information Centre (http://www.nmic.cn/) are also used. The data are generated by the Thin Plate Spline interpolation method based on *in-situ* temperature and precipitation data in China. It should be noted the DEM ancillary data is not used for data fusion in TP, because the observation stations are distributed at lower altitudes, some isotope simulations of the grid points with higher altitudes are unreasonable in the fusion methods.

Seven δ^18^O_p_ spatio-temporal fields simulated by five iGCMs (CAM2, GISS E, HadAM3, LMDZ4 and MIROC32) are used, which are selected from the SWING2 archive (available at https://data.giss.nasa.gov/swing2/). Five of eight simulations are free-running, performed following the Atmospheric Model Intercomparison Project (AMIP) protocol, using prescribed sea surface temperatures (SST) and sea ice^30,54^. The remaining three (GISS E, IsoGSM2 and LMDZ4) are nudged to constrains of large-scale atmosphere circulation, so that the dynamical fields in simulations are close to the observations^54^. In addition to SWING2 simulations, a zoomed simulation by LMDZ4, with the horizontal resolution of 50–60 km^30,55^ and nudged to reanalyses, is used. The zoomed LMDZ4 simulations used the HPC resources of IDRIS under the allocation 0292 made by GENCI. The isoGSM version 2 nudged to reanalysis with the horizontal resolution of 1° is also used^54,56,57^. Totally, nine simulations from six iGCMs are used, and detailed information about these iGCMs can be found in Table 1. In general, this study makes the maximum extent to use isotope observations and simulation in data fusion.

**Table 1:** Time periods and basic outputs information of selected iGCMs.

GCM	Simulation method	Horizontal resolution (longitude × latitude)	Time period	Key references
CAM2	Free-running	2.8° × 2.8°	1958–2003	Lee, *et al*.^111^
GISS E	Free-running and nudged by NCEP	2.5° × 2°	1969–2009	Schmidt, *et al*.^27^
HadAM3	Free-running	3.75° × 2.5°	1870–2001	Tindall, *et al*.^29^
IsoGSM2	nudged by NCEP	1° × 1°	1979–2017	Yoshimura, *et al*.^54^
LMDZ4	Free-running and nudged by ECMWF	3.75° × 2.5°	1979–2007	Risi, *et al*.^30^
	Zoomed (nudged by ECMWF)	50–60 km	1979–2017	Gao, *et al*.^55^
MIROC32	Free-running	2.8° × 2.8°	1979–2007	Kurita, *et al*.^31^

The time span of the isoscape built in this study covers the union set of all simulations ranging from 1870 to 2017. Since the temporal lengths of nine iGCM are not identical, the number of iGCM simulations used to build the isoscape varies. Specifically, for 1979–2001, a total of nine simulations from all six iGCMs in Table 1 are used; for 2002–2007, seven simulations from four iGCMs (GISS E, IsoGSM2, LMDZ4 and MIROC32) are used; and for 1969–1978, four simulations from three iGCMs (CAM2, GISS E and HadAM3) are used. For the remaining periods, there is only one simulation or two: for 1958–1968, CAM2 and HadAM3 are used; for 1870–1957, HadAM3 is used; and for 2008–2017, IsoGSM2 and zoomed LMDZ4 are used.

The stable isotope composition of precipitation is expressed in the relative permillage (‰) derived from the standard sample^58^ as:

1 $$\delta =\left(\frac{{R}_{{\rm{sample}}}}{{R}_{{\rm{V-SMOW}}}}-1\right)\times 1000\textperthousand $$

where *R* is the ratio of heavier isotope to common isotope (^18^O/^16^O), and the subscripts sample and V-SMOW represent standard sample and Vienna Standard Mean Ocean Water, respectively.

### Generation of isoscape

Generally, the generation of isoscape can be divided into five steps.

(1) Prior to generating the dataset, the inverse distance weighting (IDW) method is used to interpolate all iGCM simulations and ancillary data to observation stations.

(2) Three neural network data fusion and two bias correction methods are trained using observations and iGCM simulations for all months within a season and all stations within a sub-region. In other words, observed and simulated monthly isotopes within a season and all stations within a region are pooled to train the data fusion and bias correction methods to ensure that the model is well trained with enough samples. For the fusion methods, ancillary data are also included in the training process, which is not necessary for bias correction methods.

(3) The performance of each model is evaluated for the validation period by the cross-validation method to find the optimal data fusion and bias correction methods. Correlation coefficient (CC) and root mean square error (RMSE) are used as metrics to validate these methods for the common period of 1969–2007.

(4) All iGCM simulations and ancillary data are interpolated to the LMDZ4 zoomed grid with a spatial resolution of approximately 50 km by the IDW method.

(5) The optimal trained model and bias correction methods are applied to all grid points within a sub-region and all months within a season. Since the length of iGCM simulations is not identical, the optimal combination of data fusion and bias correction methods are used to generate the isoscape for a long period. In other words, for the common period of observations and iGCM simulations, the optimal data fusion method is used, while for the period with no observations, the bias correction methods are used.

### Neural network data fusion

The neural network is a kind of mathematical model, which imitate the behaviour characteristics of human neural network and carry out distributed parallel computing^59,60^. Performing calculations and spreading information through large numbers of interconnected neurons, neural networks are often used to describe complex relationships between inputs and outputs, or to explore the internal structure and patterns of data^61,62^. In this study, Back Propagation Neural Network (BP), Long Short-Term Memory (LSTM) Neural Network and Convolutional Neural Network (CNN) are adopted for data fusion, considering BP’s simplicity and practicality, LSTM’s advantages in time series prediction and CNN’s outstanding performance in various fields.

**Fig. 2:**
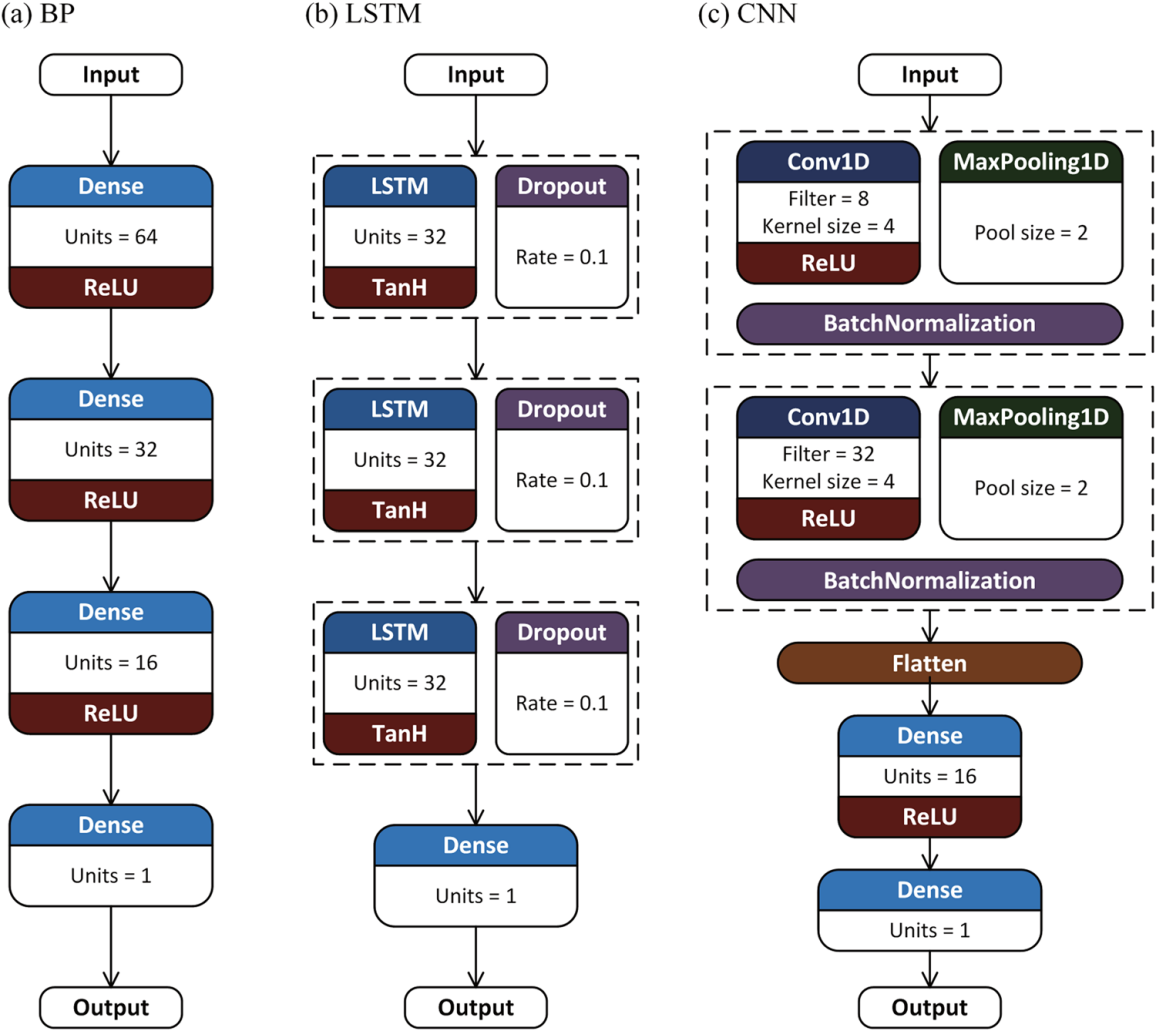
Network structure of BP, LSTM and CNN fusion method. The complete hyperparameter setting of the neural network fusion methods can be seen in Table S2.

The structure and hyperparameters of these neural network methods are carefully considered and validated. Considering that previous studies^63–66^ on hyperparameter sensitivity of neural networks have shown similar results, the hyperparameter selection scheme is determined based on these studies using a hierarchical stepwise search method to determine hyperparameter values. Specifically, the hyperparameters are divided into three parts, structural hyperparameters, sensitive algorithm hyperparameters and other algorithm hyperparameters, which are determined step by step. At each step, the performance of all hyperparameter combinations is tested using the grid search method. Referring to previous studies^45,67–69^, some conventional hyperparameter settings (such as filters are usually set to the power of 2) are considered. The details of hyperparameter selection are shown in Table 2. Furthermore, when structural hyperparameter values produce similar performance, the simpler structure (i.e., the one with fewer hyperparameters) is chosen to avoid overfitting. When algorithm hyperparameter values produce similar performance, the one that is more efficient for computing is chosen. The structures of these three neural network DFMs are presented in Fig. 2.

**Table 2:** Details of the hyperparameter selection in three neural network fusion methods.

Model	Steps	Hyperparameters	Range tested	Selected
BP	Step 1. Structural hyperparameters	Hidden layers	2, 3, 4	3
	Step 2. Sensitive algorithm hyperparameters	Learning rate	0.0001–0.005	0.005
		Batch size	10–50	20
		Dense neurons	8, 16, 32, 64	16/32/64
	Step 3. Other algorithm hyperparameters	Activation	ReLU, TanH	ReLU
LSTM	Step 1. Structural hyperparameters	LSTM layers	2, 3, 4	3
	Step 2. Sensitive algorithm hyperparameters	Learning rate	0.0001–0.005	0.001
		Batch size	10–50	50
		LSTM neurons	8, 16, 32, 64	32
	Step 3. Other algorithm hyperparameters	Time steps	1–5	2
		Dropout rate	0–0.3	0.1
		Activation	ReLU, TanH	TanH
CNN	Step 1. Structural hyperparameters	Convolutional layers	1, 2, 3	2
		Dense layers	1, 2	1
	Step 2. Sensitive algorithm hyperparameters	Learning rate	0.0001–0.005	0.0005
		Batch size	10–50	50
		Kernel size	3, 4, 5	4
	Step 3. Other algorithm hyperparameters	Filters	8, 16, 32, 64	8/32
		Dense neurons	8, 16, 32, 64	16
		Dropout rate	0–0.3	0
		Activation	ReLU, TanH	ReLU

The complete hyperparameter setting of the neural network fusion methods can be seen in Table S2.

BP, first proposed by Rumelhart, *et al*.^70^, is a multilayer feed-forward network trained by the error back-propagation algorithm. BP is one of the most widely used neural network models with high simulation accuracy for nonlinear functions. The main characteristic of BP is that the input signal is processed layer by layer from the input layer to the hidden layer and then to the output layer, and each neuron carries out the weighted sum of the input signal through the activation function. If the error between the actual output and the expected output is larger than the set value, the weight and bias of the network are continuously corrected by the backpropagation to minimize the loss function. The isotope simulations of iGCMs are selected as the input of BP and the observations as the expected output to calculate the loss function. The input layer is the corresponding input parameter, and the output layer is the fusion isotope value.

LSTM is very efficient for sequential data and is derived from Recurrent Neural Network (RNN) with memory function. RNN has a sequential feed-forward connection, so that the information of the past moment can affect the output of the present moment^71^. The traditional RNN has the problems of vanishing gradient and exploding gradient^72^. To solve these problems, the LSTM Neural Network was proposed^73^. A basic LSTM neuron usually consists of a memory cell and three gates (i.e. input gate, forget gate and output gate). Memory cells are used to store past information, realizing long-distance dependent learning of sequence features. The input gate determines which inputs are saved to the cell; the forget gate determines what information is retained from the previous moment; output gate determines what information needs to be output. In this study, a fully connected layer is added after three LSTM layers to generate fusion results. Dropout layers are applied to the three LSTM layers of the network to make the model more robust.

CNN was first proposed by LeCun^74^, for the problem of handwritten digit recognition. CNN combines three advantages of local connectivity, weight sharing and pooling. On one hand, it reduces the number of weights, making the network easy to optimize. On the other hand, it reduces the complexity of the model and alleviates the overfitting problem. It is one of the most widely used neural networks with the best performance. The convolutional layer and pooling layer of the hidden layer are the core modules of CNN. The function of the convolutional layer is to extract features of the input data by convolutional kernels. The pooling layer performs feature selection and information filtering on the feature map output by the convolution layer. In this study, CNN is mainly composed of two convolutional layers and pooling layers. Two fully connected layers are added at the end to remove the spatial topology and output the results. The convolutional layer and the fully connected layer are connected by flattening the output of the convolutional layer through the flatten layer. Batch normalization layers are inserted into the model to improve the speed, performance and stability of the neural network.

### Bias correction methods

BCMs aim to correct the mean, variance and/or quantile of the climate model time series, so that the corrected model time series can better match those of the observations^75^. In this study, two typical methods (i.e. linear scaling (LS) and distribution translation (DT)) are used to correct the bias of iGCM at the monthly timescale. These two BCMs can be classified into mean-based scaling (i.e. LS) and distribution-based correction (i.e. DT) approaches^76^. The mean-based scaling uses a constant correction factor for the entire time series, while the distribution-based approach uses correction factors that vary with the quantiles of the distribution^77^.

The LS method is the simplest bias correction method. The differences between observations and raw iGCM simulations are applied to simulations to obtain the bias-corrected isotope time series for each season and sub-region. Specifically, for a particular sub-region, the differences (defined as correction factors) in mean values between observed and simulated isotopes are first calculated at the seasonal basis using Eq. (2). The calculated correction factors are then applied to simulated isotopes for the entire period using Eq. (3).

2 $${R}_{{\rm{LS,s,sr}}}={\overline{\delta O}}_{{\rm{obs,s,sr}}}^{{\rm{ref}}}-{\overline{\delta O}}_{{\rm{raw,s,sr}}}^{{\rm{ref}}}$$

3 $$\delta {O}_{{\rm{cor,s,sr}}}=\delta {O}_{{\rm{raw,s,sr}}}+{R}_{{\rm{LS,s,sr}}}$$

where *R*_LS_ is the correction factor; $\overline{\delta O}$ is the mean value of isotope composition; the superscript *ref* represents the reference period; the subscripts *obs*, *raw* and *cor* represent observations, raw simulations and corrected simulations, respectively; and *s* and *sr* represent a specific season and a sub-region, respectively.

The implementation of the DT method is similar to the LS method. However, the differences (i.e. correction factors) between observed and simulated isotopes are calculated for each of 100 integral percentiles as shown in Eqs. (4–6), to represent the distribution for each season in each sub-region. The correction factors of grid points are obtained by interpolating or extrapolating the factors of observation stations using Eq. (5).

4 $${R}_{{\rm{DT,s,sr}}}^{{\rm{ref}}}=\delta {O}_{{\rm{obs,q,s,sr}}}^{{\rm{ref}}}-\delta {O}_{{\rm{raw,q,s,sr}}}^{{\rm{ref}}}$$

5 $${R}_{{\rm{DT,s,sr}}}^{{\rm{ref}}}\mathop{\to }\limits^{{\rm{Interpolation/Extrapolation}}}{R}_{{\rm{DT,s,sr}}}$$

6 $$\delta {O}_{{\rm{cor,s,sr}}}=\delta {O}_{{\rm{raw,s,sr}}}+{R}_{{\rm{DT,s,sr}}}$$

where the subscript *q* is a percentile for a specific season in a sub-region. Other superscripts and subscripts are the same as Eqs. (2, 3).

### Model performance

Correlation coefficient (CC) and root mean square error (RMSE) are used as metrics to quantify model performance.

7 $$CC=\frac{1}{N-1}\mathop{\sum }\limits_{i=1}^{N}\left(\frac{{O}_{i}-{\mu }_{O}}{{\sigma }_{O}}\right)\left(\frac{{S}_{i}-{\mu }_{S}}{{\sigma }_{S}}\right)$$

8 $$RMSE=\sqrt{\frac{1}{N}\mathop{\sum }\limits_{i=1}^{N}{\left({S}_{i}-{O}_{i}\right)}^{2}}$$

where *S* and *O* are the simulated and observed value, respectively; *N* is the number of samples; and *μ* and *σ* are the mean and standard deviation, respectively.

### Cross-validation experiments

In order to make full use of the data and reduce the variation of model accuracy caused by the difference between the training set and the test set, K-fold cross-validation is adopted. In the K-fold (K = 5 in this study) experiment, the data set is randomly divided into K groups, and one of them is used as the test set each time, leaving K-1 groups as the training set. To fully consider the variations of random division, K-fold cross-validation is repeated 100 times.

## Data Records

**Fig. 3:**
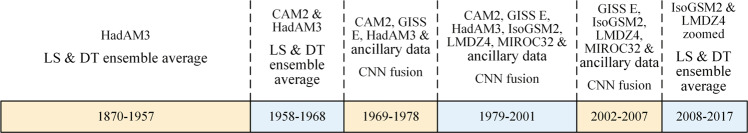
The generation mode of dataset in each period.

The dataset includes the stable oxygen isotope of precipitation for the mainland of China over the 1870–2017 period, at a spatial resolution of 50–60 km and a monthly temporal resolution. In order to make full use of observations to integrate the advantages of various iGCMs, the combination of data fusion and bias correction methods are used (as shown in Fig. 3). Specifically, (1) for the 1979–2001 period, nine simulations from six iGCMs (CAM2, GISS E, HadAM3, IsoGSM2, LMDZ4, and MIROC32) and ancillary data are fused with observations by using CNN fusion method; (2) for the 2002–2007 period, seven simulations from four iGCMs (GISS E, IsoGSM2, LMDZ4, and MIROC32) and ancillary data are fused by using CNN fusion method; (3) for the 1969–1978 period, four simulations from three iGCMs (CAM2, GISS E, and HadAM3) and ancillary data are fused by using CNN fusion method; (4) for the 1958–1968 and 2008–2017 periods, two iGCM simulations (CAM2 and HadAM3 for 1958–1968 and IsoGSM2 and LMDZ4 zoomed for 2008–2017) are corrected by using two BCMs, and ensemble mean (mean of four simulations) is then calculated; (5) for the 1870–1957period, one iGCM simulation (HadAM3) is corrected by using two BCMs, and ensemble mean (mean of two simulations) is then calculated. The dataset^78^ is freely available in Zenodo repository (10.5281/zenodo.7306199) with the format of netCDF4.

## Technical Validation

### Evaluation of bias correction and data fusion methods

**Fig. 4:**
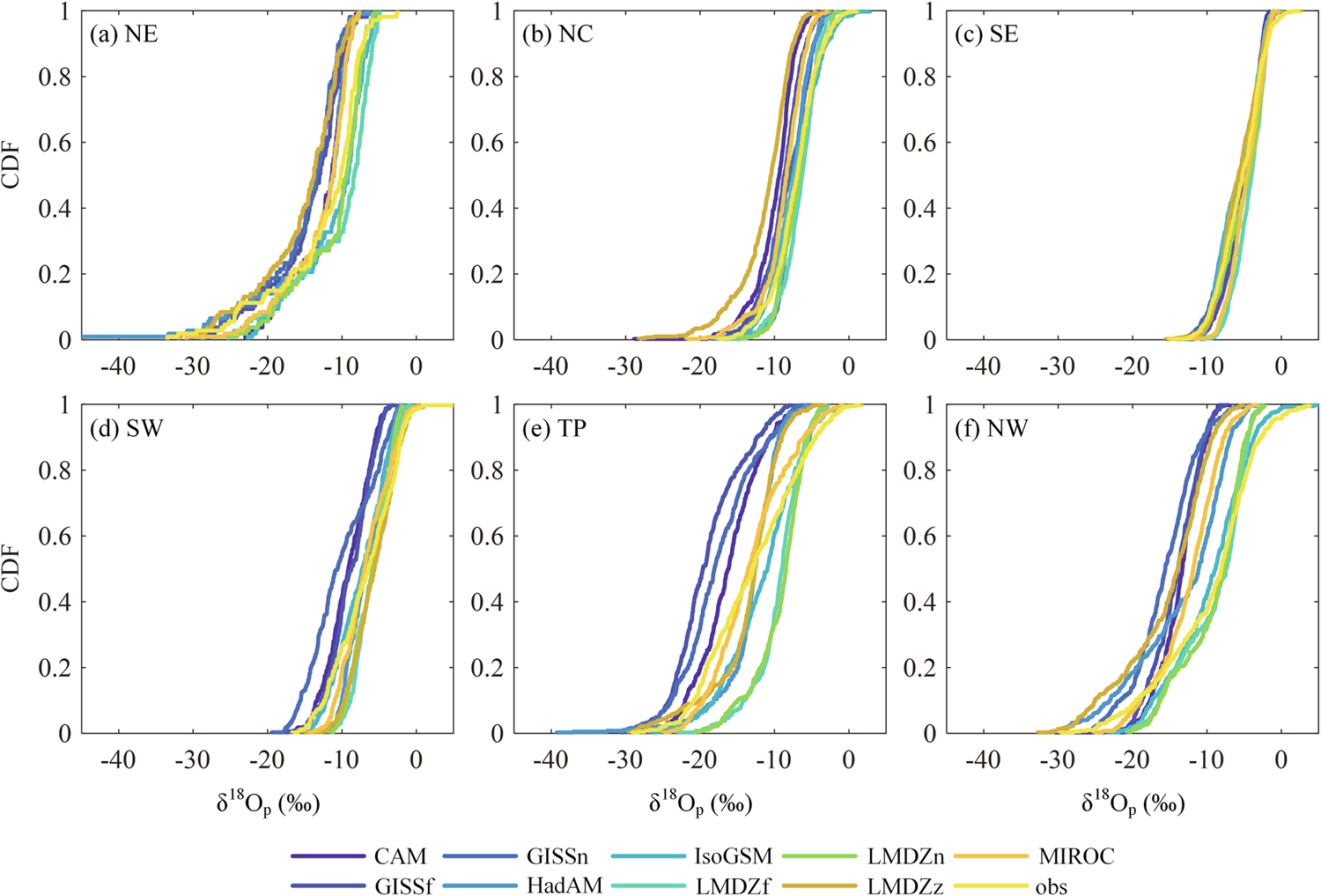
Cumulative distribution functions of δ^18^O_p_ for eight iGCM simulations in six sub-regions.

Prior to applying BCMs and DFMs to build the isoscape, the performance of iGCM simulations is evaluated by comparing gauged observations for the common period of 1969–2007. Figure 4 shows the cumulative distribution functions (CDFs) of δ^18^O_p_ for observations and iGCMs simulations in each sub-region. Generally, the CDFs of observed δ^18^O_p_ can be well represented by iGCM simulations for each sub-region, as the observed CDFs distribute in the centre of simulated ones. For specific regions, the envelope of CDFs is the narrowest for SE, indicating that iGCMs perform consistently better for this region. For NE, NC, SW and NW, the CDFs of IsoGSM2 and LMDZ4 (free and nudged) simulated δ^18^O_p_ are relatively close to the observations, while other iGCM simulations generally overestimate the δ^18^O_p_. For TP, the differences between CDFs of observed and simulated δ^18^O_p_ are the largest, indicating the prominent variability of δ^18^O_p_ simulations. This is expected, as climate models generally perform worse for TP than other regions^79,80^. For NW, the variability of δ^18^O_p_ simulations is also large. This is because, on the one hand, the sparse coverage of stations coupled with complex topography over northwest China cannot well represent the full range of precipitation isotope conditions. This can lead to biases in the distribution of observations. On the other hand, the arid northwest region is one of the most sensitive regions to climate change due to its fragile ecosystem, which affects sub-cloud evaporation and local moisture re-cycling, leading to the large uncertainty in isotope simulation between different iGCMs^23,81^.

**Fig. 5:**
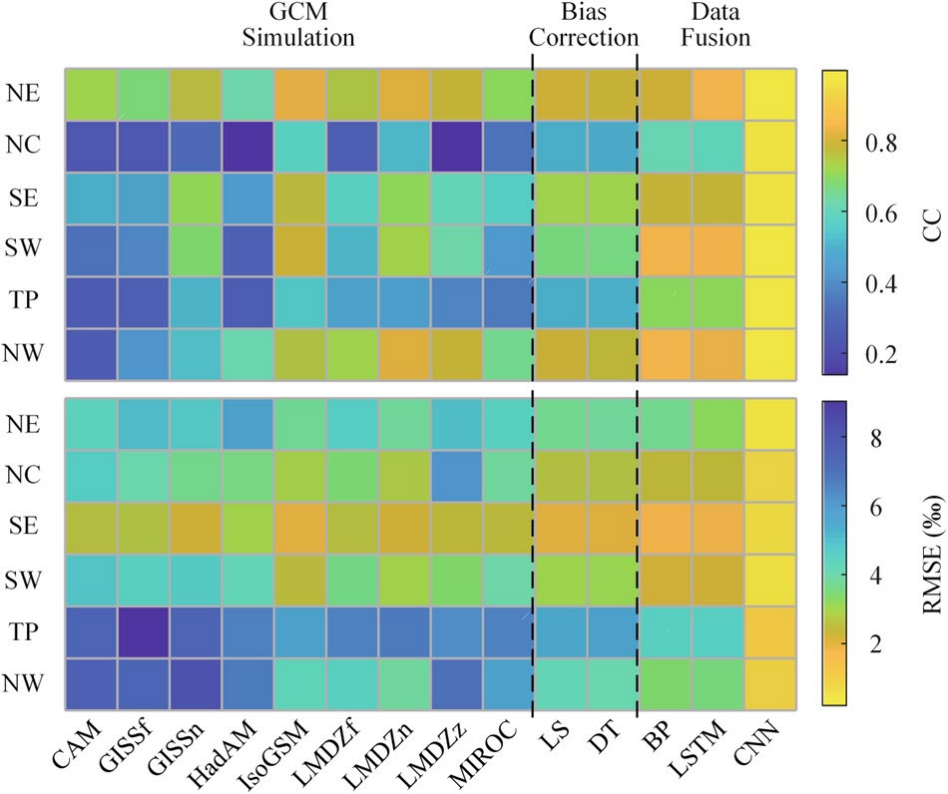
Average correlation coefficient (CC) and root mean square error (RMSE) metrics of raw, bias-corrected and fused δ^18^O_p_ in six sub-regions.

Root mean square error (RMSE) and correlation coefficient (CC) are also calculated to evaluate the accuracy of iGCM simulated δ^18^O_p_. DFMs that introduce ancillary data generally perform better than DFMs that do not introduce that, with larger CC and smaller RMSE. Therefore, ancillary data are introduced into all DFMs in this study. Figure 5 presents the RMSE and CC for raw iGCM simulations, bias corrected and fused simulations for six sub-regions over the validation periods (1979–2001). Generally, DFMs perform better than BCMs, and both perform better than raw iGCM simulations. In addition, all the simulations are correlated with the observations with CC ranging between 0.12 and 0.99.

The CC and RMSE vary considerably for raw iGCM simulations, with CC ranging from 0.12 to 0.84 and RMSE ranging from 1.9‰ to 9.1‰. The simulations of IsoGSM2, nudged GISS E and nudged LMDZ4 have the strongest correlation with the observations, and their CC is basically above 0.5, ranging from 0.30–0.84. The error of IsoGSM2 and nudged LMDZ4 is the smallest, and their RMSE ranges between 1.9‰ and 6.8‰.

Generally, the performance of LS and DT is similar, even though the LS method performs slightly better than the DT method for some regions. The CCs range from 0.49 to 0.81 for the LS and DT corrected simulations, with an average increase from 0.53 to 0.66 relative to raw simulations; the RMSEs are between 1.9‰ and 5.7‰, with an average decrease of 23.7%.

For DFMs, BP and LSTM show similar performance, while CNN consistently performs the best. The CC of CNN-generated simulations is all greater than 0.97, increasing from 0.53 to 0.99 on average compared with the raw simulations. The RMSE of CNN-generated simulations is all smaller than 1.1‰, showing an 84.3% reduction relative to the raw simulation on average.

Figure 5 shows that CC and RMSE of simulations in different sub-regions are quite different. For all raw, fused, and bias corrected simulations, CC is smaller for NC and TP while RMSE is larger for TP and NW than other sub-regions. For DFMs, especially CNN, the differences of CC and RMSE between different regions are smaller.

Generally, all simulations perform worse in NC, TP and NW than in other sub-regions. The poor simulation performance in NC may be due to complex air mass movements^82,83^, which are difficult to be accurately simulated by iGCMs. The poor simulation performance in TP and NW may be due to the fact that GCMs cannot accurately describe the atmospheric physical process and simulate precipitation and other meteorological factors in these regions^84–86^.

**Fig. 6:**
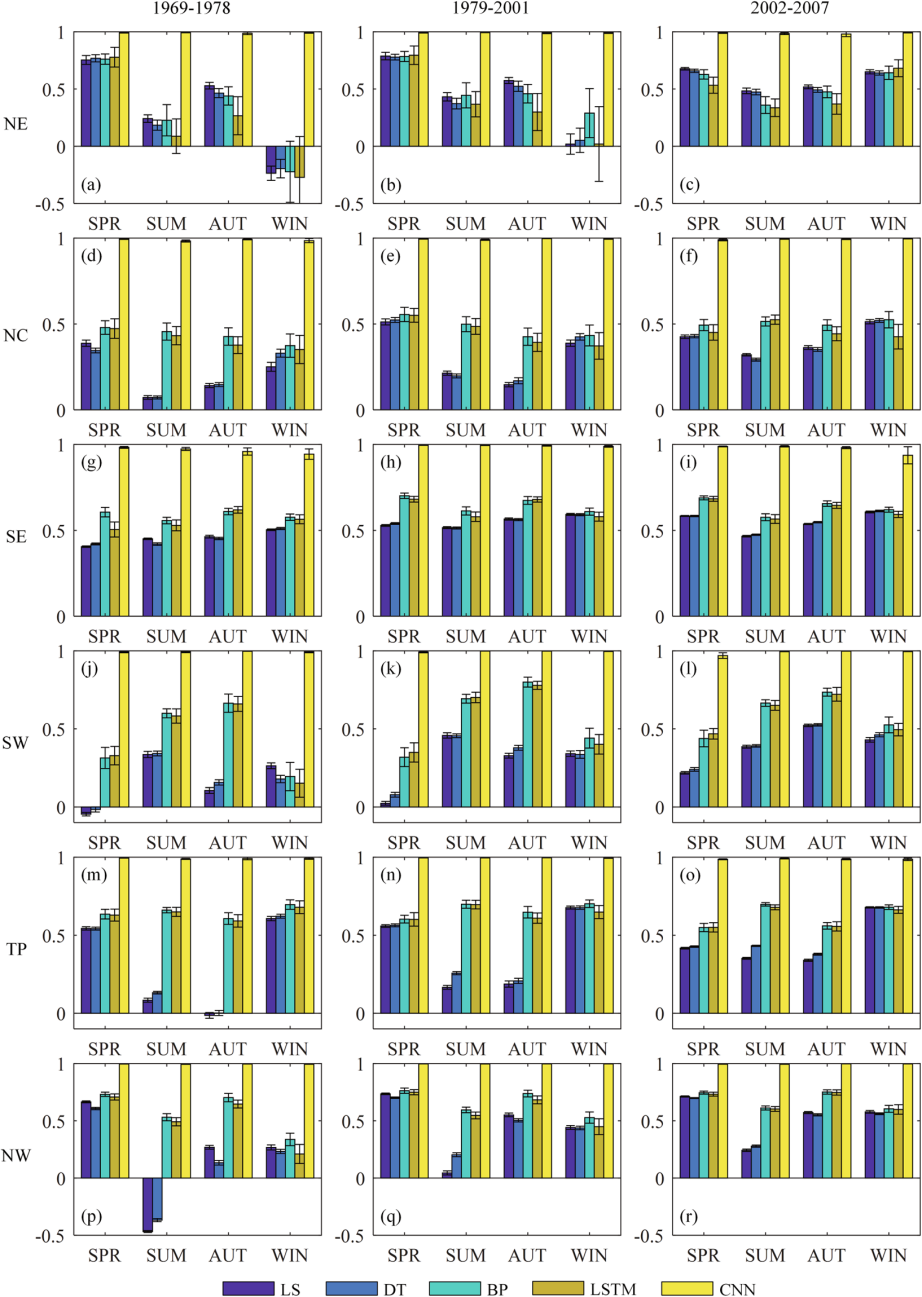
Seasonal average results of correlation coefficient (CC) metrics for BCMs and DFMs in six sub-regions. The whiskers denote +/− one standard deviation.

**Fig. 7:**
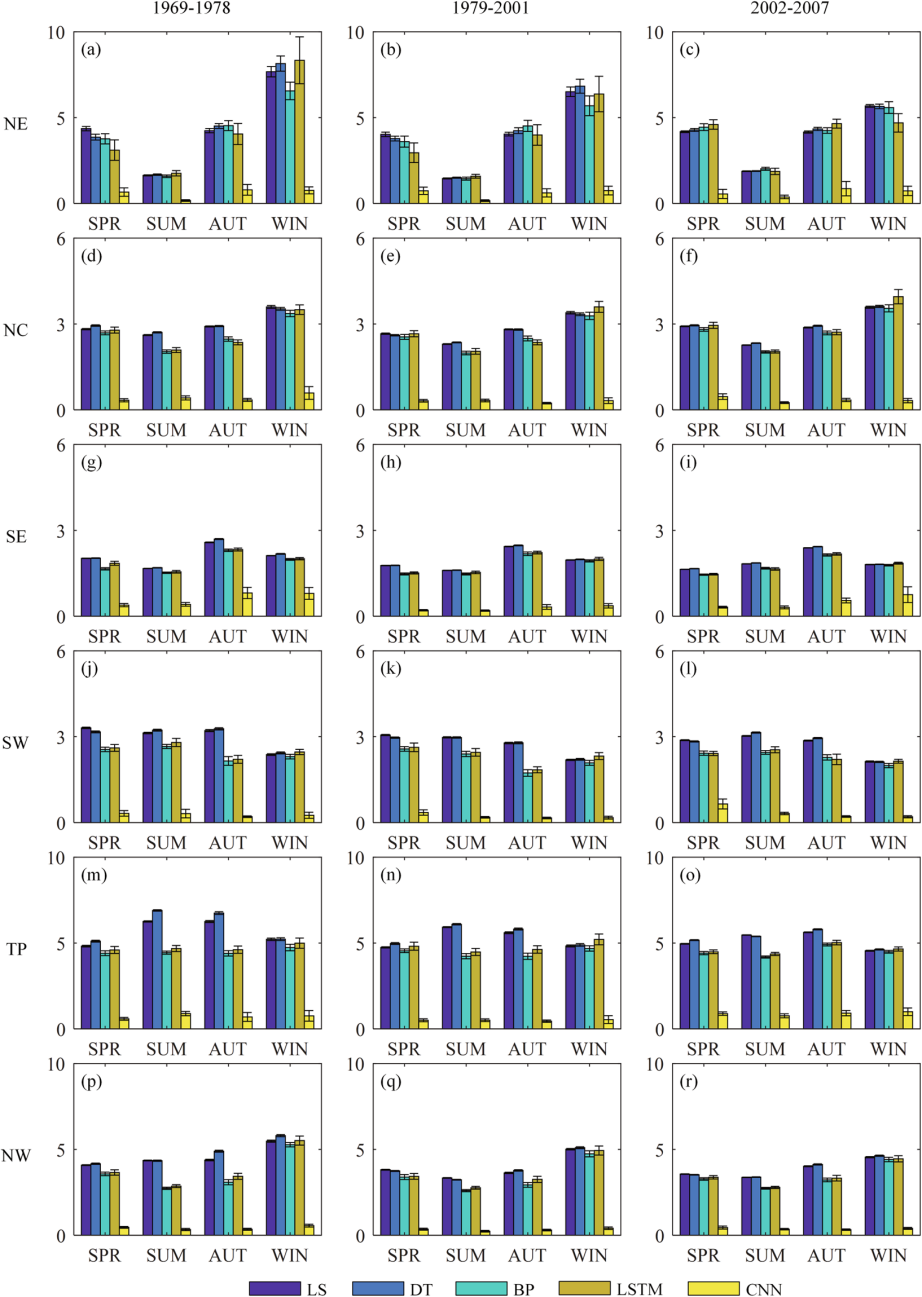
Seasonal average results of root mean square error (RMSE) metrics (‰) for BCMs and DFMs in six sub-regions. The whiskers denote +/− one standard deviation.

The performance of BCMs and DFMs is also evaluated for three periods (1969–1978, 1979–2001 and 2002–2007) and six sub-regions on a seasonal basis. The seasonal average CC and RMSE for two BCMs and three neural network DFMs are presented in Figs. 6, 7. The seasons are divided into spring (SPR), summer (SUM), autumn (AUT) and winter (WIN), respectively defined as March-May, June-August, September-November, and December-February. Generally, all BCMs and DFMs perform very similarly for all three periods.

As for CC, the simulations in the northern region (NE, NC, and NW) show strong correlations with observations in spring, while those in the southern region (SE, SW) show strong correlations in summer and autumn. The correlation of CNN fusion simulations is significantly higher than that of the other methods, with CC being mostly above 0.95. The correlation of BP and LSTM fusion simulations is slightly higher than that of LS and DT corrected simulations, with CC being mostly between 0.3 and 0.7, varying with sub-regions and seasons. The BP and LSTM fusion methods perform slightly worse in NE, but better in other sub-regions, compared with BCMs.

As for RMSE, the errors of simulations in NE, TP and NW are relatively large, with an average error of about 5‰, while those in other sub-regions have an average error of about 3‰ or less. The northern region shows a small error of about 2‰ in summer, except for NW with a larger error of 3‰; while the southern region shows relatively small seasonal differences in error, mostly ranging between 1‰ and 3‰. On the whole, the DFMs perform better than the two BCMs. The errors of CNN simulations are the smallest in all regions and seasons, which are mostly smaller than 1‰. The errors of BP and LSTM simulations are slightly smaller than those of LS and DT simulations.

The plotted +/− one standard deviation shows the dispersion degree of CC and RMSE for the simulated results of all bias correction and data fusion methods over 100 trials. The standard deviations of CC and RMSE calculated by LS and DT corrected simulations are smaller, while those calculated by BP, LSTM and CNN fused simulations are relatively larger. Generally, the standard deviation of CC and RMSE calculated by the CNN fused simulations is the smallest among fusion methods. It can be considered that the correction methods show smaller uncertainties than the fusion methods in terms of CC and RMSE. This is as expected, since the simulations of DFMs show uncertainties brought by the neural network itself, in addition to the uncertainties brought by cross-validation. Furthermore, CNN fusion methods show smaller uncertainties than the other two fusion methods.

**Fig. 8:**
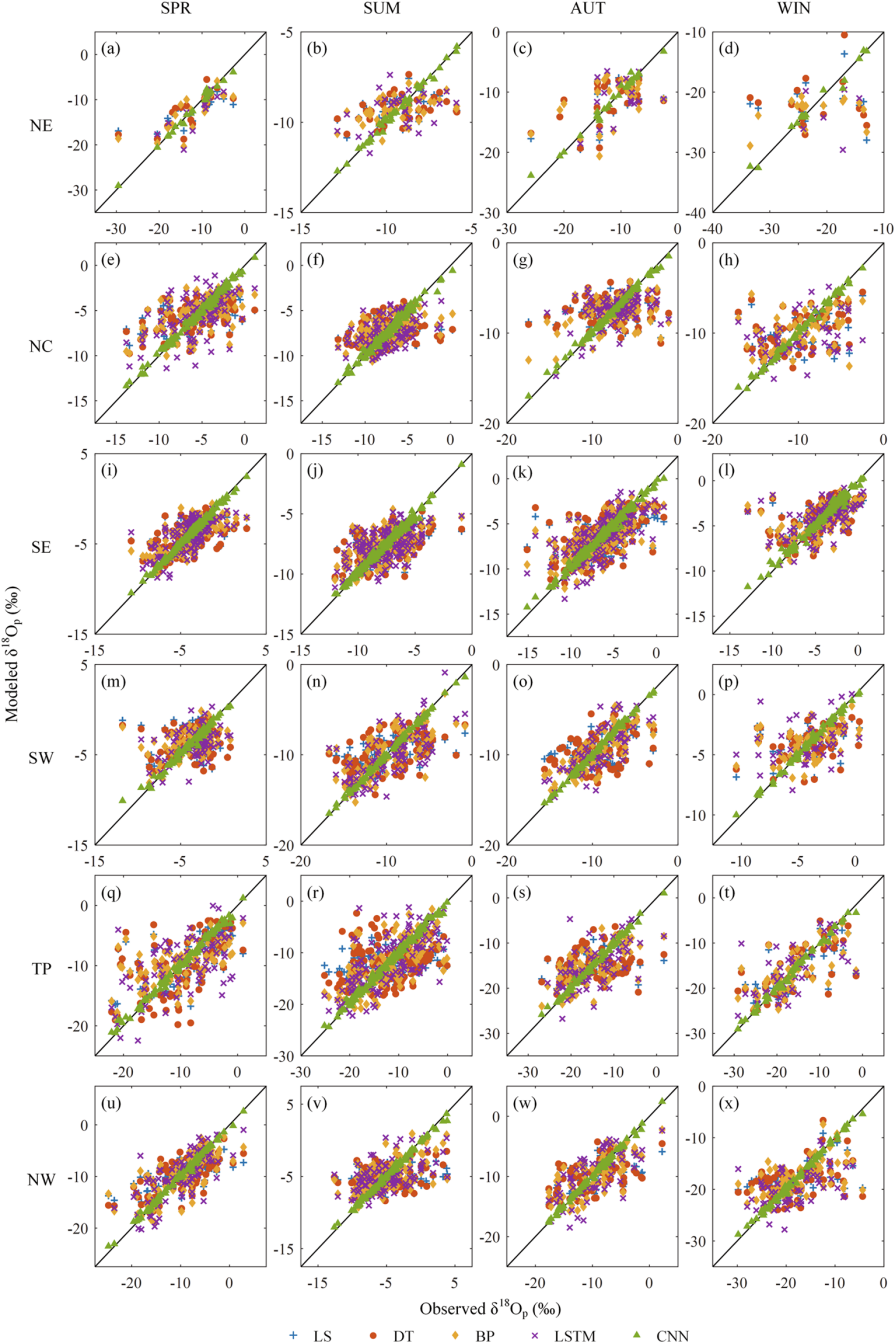
Scatter plots of seasonal δ^18^O_p_ from bias-corrected and fused output against observations in six sub-regions.

The above results show that the CNN fusion method consistently performs better than the other methods. To further confirm this conclusion, scatter plots of fused and corrected against observed δ^18^O_p_ are presented in Fig. 8 for the 1979–2001 period. The overall mean CC and RMSE corresponding to the figure are shown in Table 3. It can be seen that the CNN fusion method shows a stronger correlation with the observations than the other fusion methods and BCMs. The CNN fused δ^18^O_p_ consistently shows the largest CC and smallest RMSE, showing a strong positive linear correlation with the observations with CC being almost all larger than 0.99.

**Table 3:** Correlation coefficient (CC) and root mean square error (RMSE, ‰) metrics corresponding to the Fig. 8.

Sub-region	Season	LS		DT		BP		LSTM		CNN	
		CC	RMSE	CC	RMSE	CC	RMSE	CC	RMSE	CC	RMSE
NE	SPR	0.791	4.058	0.774	3.769	0.784	3.642	0.799	3.030	0.996	0.628
	SUM	0.433	1.457	0.379	1.497	0.448	1.445	0.361	1.507	0.995	0.156
	AUT	0.578	4.030	0.530	4.203	0.468	4.513	0.331	4.059	0.993	0.623
	WIN	0.024	6.508	0.067	6.834	0.277	5.696	0.079	6.289	0.992	0.770
NC	SPR	0.510	2.665	0.521	2.612	0.556	2.550	0.556	2.654	0.995	0.315
	SUM	0.213	2.297	0.199	2.348	0.504	1.979	0.485	1.991	0.991	0.329
	AUT	0.151	2.806	0.167	2.800	0.422	2.509	0.396	2.382	0.997	0.237
	WIN	0.386	3.388	0.425	3.337	0.434	3.302	0.376	3.571	0.997	0.310
SE	SPR	0.529	1.771	0.540	1.777	0.701	1.474	0.683	1.519	0.995	0.208
	SUM	0.520	1.596	0.516	1.607	0.616	1.472	0.576	1.547	0.995	0.197
	AUT	0.568	2.432	0.567	2.468	0.674	2.182	0.680	2.225	0.994	0.355
	WIN	0.597	1.958	0.593	1.980	0.607	1.938	0.579	1.999	0.990	0.372
SW	SPR	0.021	3.051	0.078	2.953	0.325	2.568	0.346	2.560	0.993	0.364
	SUM	0.462	2.970	0.456	2.967	0.696	2.387	0.704	2.466	0.999	0.186
	AUT	0.330	2.771	0.380	2.791	0.801	1.728	0.774	1.778	0.998	0.176
	WIN	0.343	2.189	0.339	2.206	0.442	2.075	0.413	2.319	0.997	0.199
TP	SPR	0.560	4.742	0.563	4.977	0.619	4.476	0.643	4.495	0.997	0.467
	SUM	0.169	5.926	0.260	6.090	0.709	4.171	0.703	4.483	0.996	0.514
	AUT	0.189	5.585	0.211	5.791	0.714	3.884	0.667	4.216	0.998	0.427
	WIN	0.676	4.836	0.676	4.887	0.697	4.705	0.645	5.307	0.996	0.639
NW	SPR	0.738	3.799	0.702	3.746	0.763	3.389	0.749	3.426	0.998	0.402
	SUM	0.044	3.341	0.205	3.231	0.596	2.594	0.545	2.748	0.998	0.282
	AUT	0.553	3.628	0.506	3.766	0.734	2.957	0.684	3.250	0.997	0.323
	WIN	0.441	5.015	0.437	5.090	0.533	4.730	0.449	5.030	0.997	0.448

Based on the above performances of the correction and fusion methods, the best combination to build the dataset can be determined. Since the CNN fusion method consistently performs better than other methods, the CNN was used for the common period of all climate simulation and observations, while the bias correction methods were used for the periods with only one or two climate simulations, and with no observations. The specific generation mode of the dataset for each period can be found in the Data Records section.

To evaluate the dataset for all stations, CC and RMSE are calculated for δ^18^O_p_ series between observations and raw iGCM simulations, and between observations and the fused isoscape for all stations over the common period (Tables S3-4). Also, the distributions of these CCs and RMSEs are shown in the form of histogram (Fig. S1). The results show that the built isoscape performs excellent for the vast majority of stations, with much larger CCs and smaller RMSEs than iGCM simulations. Specifically, the CCs between the isoscape simulations and observations are larger than 0.8 for 77.6% of stations and larger than 0.9 for 57.0% of stations. The RMSEs between the isoscape simulations and observations are smaller than 3‰ for 80.4% of stations and less than 2‰ for 56.1% of stations.

**Fig. 9:**
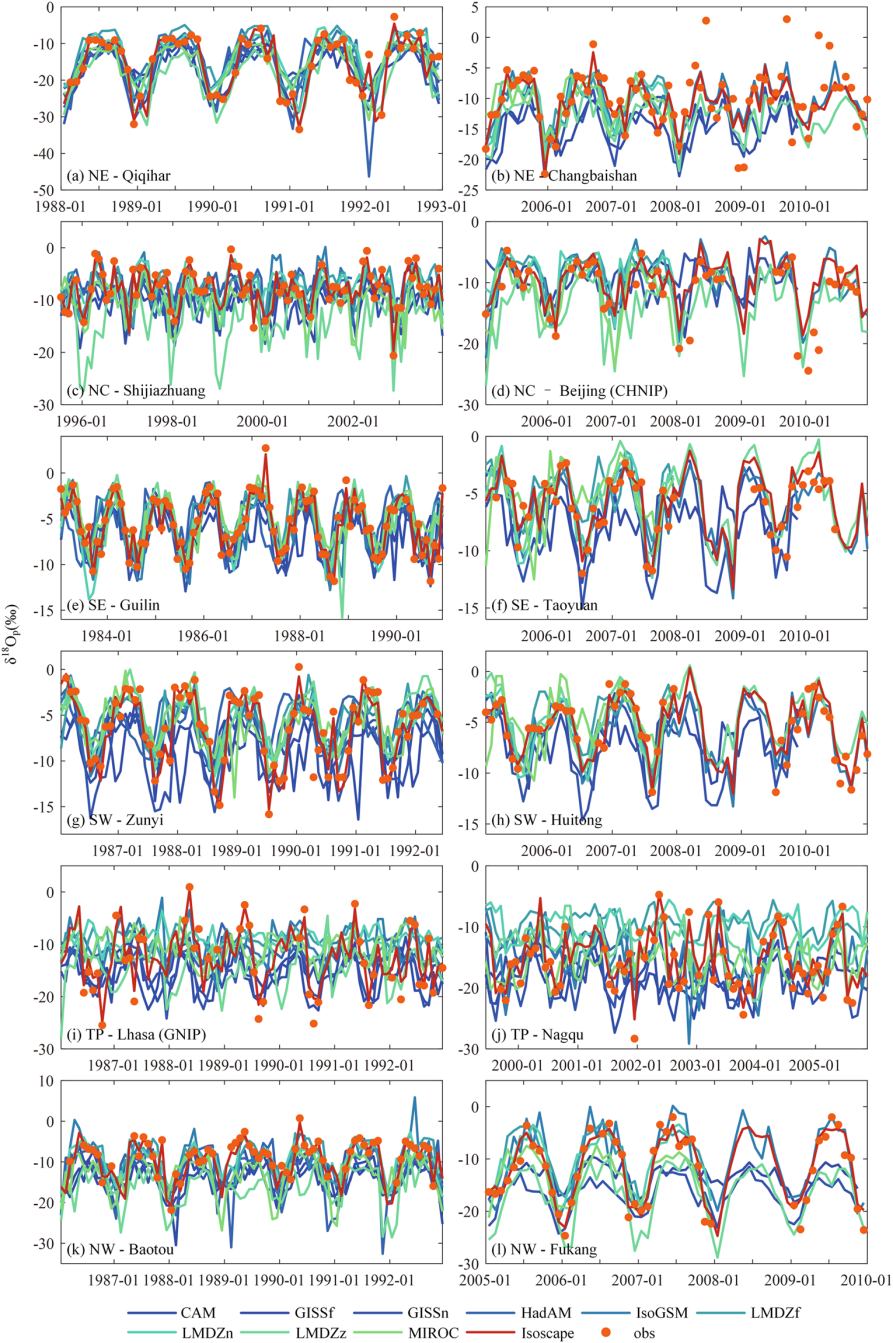
Time-series comparisons of δ^18^O_p_ among the built isoscape, iGCM simulations, and *in-situ* observations at selected stations in each sub-region.

To further demonstrate the dataset quality, twenty stations with appropriate lengths of observation are selected with two stations randomly selected from each sub-region. The time series of δ^18^O_p_ are plotted for observations, iGCM simulations, and the generated isoscape (Fig. 9). Figure 9 shows that the variations of fused δ^18^O_p_ time series show consistent patterns with observations, and it also performs much better than raw iGCM simulations. In particular, for the period before 2007, the CNN model integrates the advantages of various simulations and captures most features of the observed data. These results generally prove the reliability of fused isoscape.

Fig. S2 shows the spatial distribution of monthly mean observed, fused, and best-performing iGCM raw δ^18^O_p_ for their common period (i.e. 1979–2007). The spatial pattern presented by the isoscape shows the best consistency with observations. The strength of the CNN model has been demonstrated, which can make good use of the advantages of each simulation to accurately capture the characteristics of observations. For example, nudged LMDZ4 model shows a strong ability to reproduce the spatial distribution of δ^18^O_p_ for the eastern region in summer and autumn, but a slightly poor performance in the Qinghai-Tibet Plateau. The built isoscape combines nudged LMDZ4, GISS E, IsoGSM2 and other simulations, which show reasonable performance for the Qinghai-Tibet Plateau, and well reproduces the spatial distribution of δ^18^O_p_ for the mainland of China. In addition, the spatial and seasonal variability presented in the built isoscape is consistent with the monthly isoscape (C-Isoscape) created by Wang, *et al*.^87^ based on regionalized fuzzy clustering, with only minor differences that may be due to different data or methods. Compared with C-Isoscape, the δ^18^O_p_ of our isoscape is generally lower in the Qinghai-Tibet Plateau, and the seasonal variation of δ^18^O_p_ in northwest China is smaller.

### Spatial variability of precipitation oxygen isotope

**Fig. 10:**
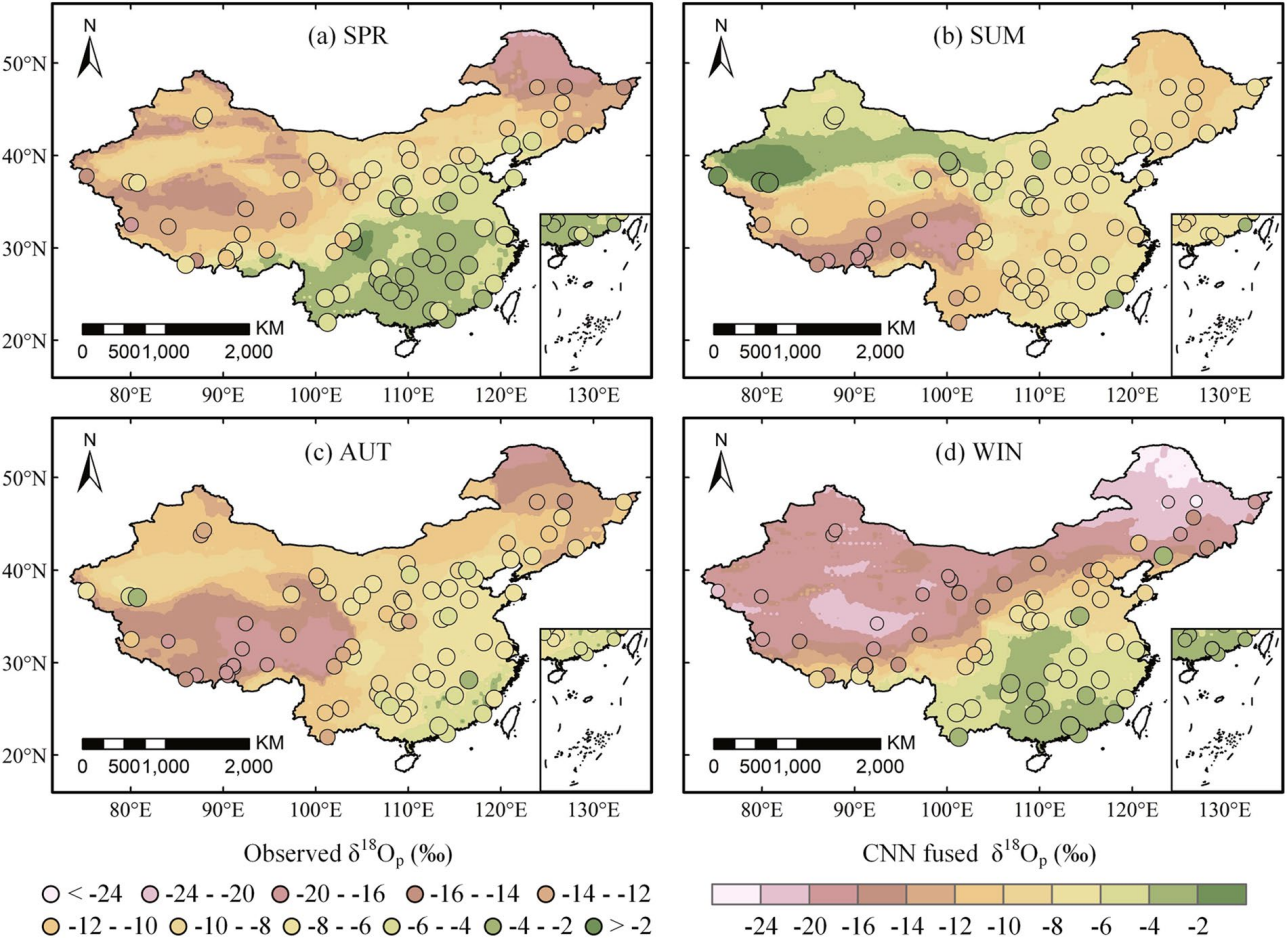
Seasonal averaged observations and CNN fused simulations of δ^18^O_p_ in the mainland of China.

To further evaluate the isoscape fused by the CNN method, the observations and the isoscape simulations are compared at the seasonal scale for analysing the spatial variability. Figure 10 presents the spatial variability of mean δ^18^O_p_ for observed and fused data for the 1969–2007 period. Generally, the CNN fused δ^18^O_p_ shows similar spatial distribution to observations for all four seasons.

In NE, δ^18^O_p_ decreases with increasing latitude for all seasons, and its spatial variation is basically parallel to the latitude, which reflects the latitude effect^88^. Indeed, most of the water vapour in the atmosphere is formed at low latitudes, and Rayleigh distillation continuously depletes the residual water vapour as air masses move toward higher latitudes, thus depleting the δ^18^O_p_ of the residual water vapour and thus of the rain forming in clouds.

In SE and SW, δ^18^O_p_ decreases from the southeast coast to inland. This phenomenon is consistent with the continental effect. As water vapour transfers from the ocean to the interior of the continent, precipitation is formed along the way. The separation process of heavy isotopes takes place preferentially than that of light isotopes, which leads to the gradual dilution of heavy isotopes in the cloud, and thus makes the proportion of heavy isotopes in the subsequent precipitation lower.

The δ^18^O_p_ in TP is low in general except for the southeast corner, which is mainly due to the special effect of large landforms^89^. The low δ^18^O_p_ in TP is mainly due to its high altitude, with an average altitude being above 4,000 m. The moisture in the air mass is gradually removed during the orographic uplift, with heavy isotopes preferred to be removed during the condensation process, which leads to the dilution of heavy isotopes in water vapour^88^. Higher δ^18^O_p_ in the southeast corner of TP indicates closer vapour sources such as the Bay of Bengal and the Arabian Sea in the Indian Ocean. Due to the terrain barrier of the Himalayas, most of the water vapour can only pass through its southeast corner, along the valley of the major rivers (Nujiang River, Jinsha River, etc.) into the plateau, or through the Yarlung Zangbo River valley into the plateau^90^.

The δ^18^O_p_ in NW is lower than that in the southern region, but higher than that in NE and TP. This is because NW is far away from the ocean and has a dry climate, so the amount of heavy isotope in water vapour from the ocean is limited. However, a large part of water vapour to generate precipitation in NW comes from terrestrial evaporation^91^. The δ^18^O_p_ in surface water in the arid area is high, resulting in high δ^18^O_p_ in evaporation water vapour and heavy isotope enrichment in precipitation. Another process is the re-evaporation of raindrop in arid climate, enriching heavy isotopes in precipitation water^92^. Under the joint control of both, the δ^18^O_p_ in this sub-region varies greatly. In the southern part of the Taklimakan Desert, Xinjiang, δ^18^O_p_ is obviously higher. This is because, the Taklimakan Desert is located in the heart of Eurasia, and it is surrounded by high mountains and has extremely low rainfall^93^. In the southern part of the desert, there is more precipitation in the Kashi-Hotan line, and the water vapour mainly comes from the evaporation of local lakes and rivers^94^, so the ratio of isotopes in precipitation is high.

The seasonality of δ^18^O_p_ varies in sub-regions and is influenced by various factors. For NE and NC, δ^18^O_p_ is lower in winter than in other seasons. NE and NC are influenced by westerly wind and polar continental air mass constantly, with no convergence or strong convection with isotope-depleted air mass. Compared with Pacific air mass, westerly wind and polar air mass are drier and have higher δ^18^O_p_^95^. The seasonal distribution pattern of δ^18^O_p_ in NE and NC is consistent with the temperature effect^96^. Although the amount effect is not significant at the annual scale in these regions, it cannot be ignored in the wet season^97^. In particular, the maximum value of δ^18^O_p_ is observed in spring for NC, when a large part of the precipitation vapour comes from local re-evaporation. The temperature effect is also reflected in NW. Due to the long-term influence of continental air mass, the temperature difference between winter and summer is large, and the δ^18^O_p_ changes synchronously with temperature in these two seasons.

For SE and SW, δ^18^O_p_ is lower in summer than in other seasons. The climate features of SE and SW are related to the deep convection driven by the East Asian monsoon^98^, which brings water vapour from the Pacific Ocean to eastern China and dominates the sub-regions in summer. Air masses from the Pacific Ocean are more isotopically depleted than those from SE and SW^83,89^, so convergence with the Pacific depleted air masses will dilute the isotopic content of precipitation in SE and SW. Therefore, although the temperature in summer is generally higher, depletion of δ^18^O_p_ is usually larger during the monsoon season than winter season. The effect of surface temperature on isotopic fractionation during precipitation is masked by the effect of precipitation amount^90^. The temporal distribution pattern of δ^18^O_p_ in SE and SW is influenced by heavy monsoon precipitation and follows the amount effect^88,96^.

In TP, δ^18^O_p_ is positively correlated with temperature in the non-monsoon region (northern part of the plateau), with high δ^18^O_p_ in summer and low in winter, reflecting the temperature effect. For the monsoon region (southern part of the plateau), δ^18^O_p_ is high in winter and spring and low in summer and autumn, which is obviously influenced by marine air mass and shows obvious amount effect. These results are similar to previous studies^48,99^.

### Temporal variability of precipitation oxygen isotope

**Fig. 11:**
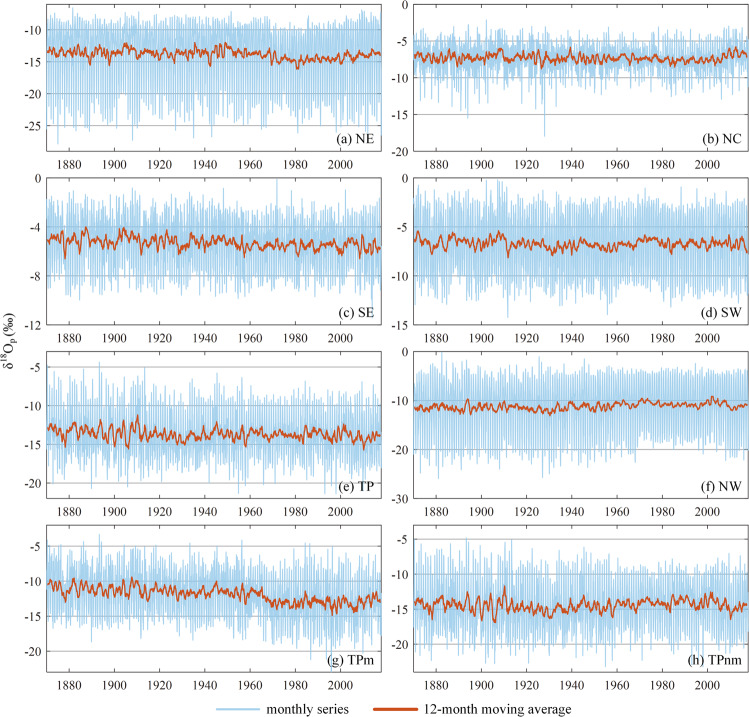
Monthly time series of the generated δ^18^O_p_ (‰) and their 12-month moving average in eight sub-regions from 1870 to 2017. (g) shows the monsoon region of TP. (h) shows the non-monsoon region of TP.

As mentioned earlier, the isoscape is generated by a combination of bias correction and data fusion methods for the 1870–2017 period. The CNN fusion is used for 1969–2007, and BCMs (mean of LS and DT) are used for the rest of the period. Figure 11 shows the monthly time series of generated δ^18^O_p_ and their 12-month moving averages for eight sub-regions over the 1870–2017 period. TP is divided into monsoon and non-monsoon regions, according to the research of Yu, *et al*.^99^. In our study, the region with significant correlations between δ^18^O_p_ and temperature is the non-monsoon region, while the rest is the monsoon region.

The Mann-Kendall tests show that the δ^18^O_p_ significantly increased in NE and NC for the recent 40 years at the P = 0.01 level. These two regions are consistent with the temperature effect, and it can be inferred that the temperature of these sub-regions has a rising trend during this period. This has been proved in many studies. For example, the studies of Ren, *et al*.^100^ and Ding, *et al*.^101^ have shown that the temperature in China has had a rising trend in recent years, especially in northeast, north and northwest China. A slight upward trend is also observed in NW from the 1930s to the 1970s (significant at the 0.1 level). The temperature effect is more significant in inland areas at middle and high latitudes. In winter, NW is mainly controlled by the westerlies, and the amount effect can be ignored^102,103^. Therefore, the temperature effect in NW is more significant in winter. From 2001 to 2012, the δ^18^O_p_ values in NW showed a decreasing trend, mainly in winter. Some studies have shown that the temperature in NW during this period is consistent with the global land warming hiatus phenomenon, and even shows obvious cooling, especially in winter^104,105^.

The δ^18^O_p_ in SE presents a gentle decline trend for the past 80 years (significant at the 0.05 level), indicating that precipitation has an upward trend in this period, since the δ^18^O_p_ conforms to the rule of amount effect in this region. While the δ^18^O_p_ in SW shows no significant trend in recent years, which indicates no significant trend in precipitation. These trends are consistent with existing researches that precipitation increased in the east coast and northwest of China, decreased in the north and northeast, and showed no significant changes in the southwest^106,107^.

There is no significant trend for δ^18^O_p_ in TP. However, δ^18^O_p_ shows a significant decreasing trend for the monsoon region of TP from the 1950s to the 2000s (significant at the 0.01 level), while it shows a significant increasing trend for the non-monsoon region (significant at the 0.05 level). This is because the non-monsoon region shows the temperature effect, while the monsoon region shows the amount effect, which is consistent with the increasing trend of temperature and precipitation in the Qinghai-Tibet region in recent years^48,99^. Overall, the changing trend of temperature and precipitation derived from the isotope effect analysis is consistent with that analysed directly using temperature and precipitation data in the mainland of China. This further proved the reasonable performance of the isoscape built in this study.

### Summary

Long-time sequences of δ^18^O_p_ are of great significance for hydrological and meteorological studies. In view of the lack of long and reliable δ^18^O_p_ datasets in China, this study generates a new dataset by integrating multi-iGCM data to overcome the limitations of short duration and uneven distribution of observed data. This dataset contains monthly δ^18^O_p_ over the mainland of China for the 1870–2017 period with a spatial resolution of 50–60 km. The dataset from 1969 to 2007 is generated by using the CNN fusion method, when the observed time series and multiple iGCM simulations are available. For other periods, it is generated by bias correcting iGCMs simulations. Two BCMs (i.e. LS and DT) with similar performances are used to produce the ensemble mean. Prior to building the isoscape, the performance of two BCMs (LS and DT) and three DFMs (BP, LSTM and CNN) is evaluated using RMSE and CC as criteria. The results show that the CNN fusion method consistently performs the best for all sub-regions in China, and BP and LSTM fusion methods perform slightly better than LS and DT (BCMs). The performance of the LS and DT methods is similar. In terms of spatial distribution and temporal variability of δ^18^O_p_, the generated data show very similar spatial distributions to observations, and the temporal trend of δ^18^O_p_ is consistent with the observed changes in precipitation and temperature for different regions in China. All these show that the built isoscape is reliable and useful to extend the time and space of observations in China.

## Usage Notes

### Advantages and limitations

The generated isoscape dataset has high spatio-temporal resolution and a long series covering 1870–2017. Compared with the existing iGCMs, the isoscape has high quality and stability for a large region in China at the monthly scale. Benefiting from the characteristics of optimal neural network and bias correction methods, the isoscape makes full use of observations to integrate the advantages of various iGCMs. In other words, by using the combination of data fusion and bias correction methods, all observations and iGCM simulations are used to the utmost extent to ensure the highest accuracy throughout the entire time period. Studies have shown that the CNN model has strong abilities for generalization and information synthesis^61,108^, while the bias correction methods have commonly used in climate change studies. Moreover, the hybrid generation method of the isoscape has the characteristics of high accuracy and simplicity, which can be easily extended to the generation of isoscape datasets in other regions. Even though the methods used in this study have been widely used, this is the first time to generate a high-quality isoscape with a long time period in China. The generated isoscape would be very used for hydro-meteorological studies. However, it should be noted that the isoscape may be more reliable for the common periods of most iGCMs (1969–2007), but mediocre for other periods. What’s more, affected by the data quality and representativeness of observation stations, the accuracy of the isoscape in some regions still needs to be improved. It is believed that this problem will be solved as observed data become more abundant.

### Data applications

Based on this built isoscape, the physical mechanisms driving the spatio-temporal variation of δ^18^O_p_ can be deeply explored. This dataset is useful for tracing atmospheric and hydrological processes. It can be used to study the effect of meteorological variables and air mass trajectory on stable isotope distribution, and quantify the source and fate of moisture^55,109,110^. For example, over East Asia, where the length of observed isotope data is short, or over the Tibetan Plateau, where data are unevenly distributed, the influence of climate change on moisture source and contribution can be studied based on this long series precipitation isoscape. The isoscape can also be used with regional climate models through data assimilation. For example, the precipitation isoscape can be combined with the physical constraints of regional climate models to reconstruct hydrological and climatic elements such as water vapour and precipitation. It can be a useful attempt to advance the study of climatic and hydrological data.

## Supplementary information

Supplementary information

## Data Availability

The codes for two bias correction methods (LS and DT) and three neural network data fusion methods (BP, LSTM and CNN) are available at 10.5281/zenodo.7306199. The codes were programmed using MATLAB version 2022a and Python 3.8.
