# Supplementary material for: A century and a half precipitation oxygen isoscape for China generated using data fusion and bias correction
Source: Sci Data. 2023 Apr 6;10:185. doi: 10.1038/s41597-023-02095-1 (PMC10079680; doi:10.1038/s41597-023-02095-1)
Supplement: Supplementary file 1 — Supplementary information [file 41597_2023_2095_MOESM1_ESM.pdf]

Supplemental information for

A century and a half precipitation oxygen isoscape for China generated using data fusion and bias correction

Jiacheng Chen[1, 2], Jie Chen[1, 2], Xunchang J. Zhang[3], Peiyi Peng[4], Camille Risi[5]

1. State Key Laboratory of Water Resources & Hydropower Engineering Science, Wuhan University, Wuhan, 430072, China
2. Hubei Key Laboratory of Water System Science for Sponge City Construction, Wuhan University, Wuhan, 430072, China
3. USDA-ARS Oklahoma and Central Plains Agricultural Research Center, 7207W. Cheyenne St., El Reno, OK 73036, USA
4. Chongqing Southwest Research Institute for Water Transport Engineering, Chongqing Jiaotong University, Chongqing, 400016, China
5. Laboratoire de Meteorologie Dynamique, IPSL, CNRS, Ecole Normale Superieure, Sorbonne Universite, PSL Research University, Paris, France

corresponding author(s): Jie Chen (jiechen@whu.edu.cn)

Table of Contents

SUPPLEMENTARY FIGURES **3**

Figure S1. Histograms of correlation coefficients (CCs) and root mean square errors (RMSEs) between $\delta^{18}O_p$ observations and raw iGCM simulations and between observations and the fused isoscape for all stations. 3

Figure S2A. Spatial distributions of $\delta^{18}O_p$ in each month for the period of 1979-2007 as obtained from observations (circles), the built isoscape (left column), and better-performing iGCMs (right three columns). 4

Figure S2B. Spatial distributions of $\delta^{18}O_p$ in each month for the period of 1979-2007 as obtained from observations (circles), the built isoscape (left column), and better-performing iGCMs (right three columns). 5

SUPPLEMENTARY TABLES **6**

Table S1. Detailed information and sources for each observation site. 6

Table S2. Complete hyperparameter settings of the neural network fusion methods. 9

Table S3. Correlation coefficient (CC) metrics of $\delta^{18}O_p$ series between observations and iGCM simulations and the built isoscape at all stations. 10

Table S4. Root mean square error (RMSE, ‰) metrics of $\delta^{18}O_p$ series between observations and iGCM simulations and the built isoscape at all stations. 13

REFERENCES **16**

Supplementary Figures

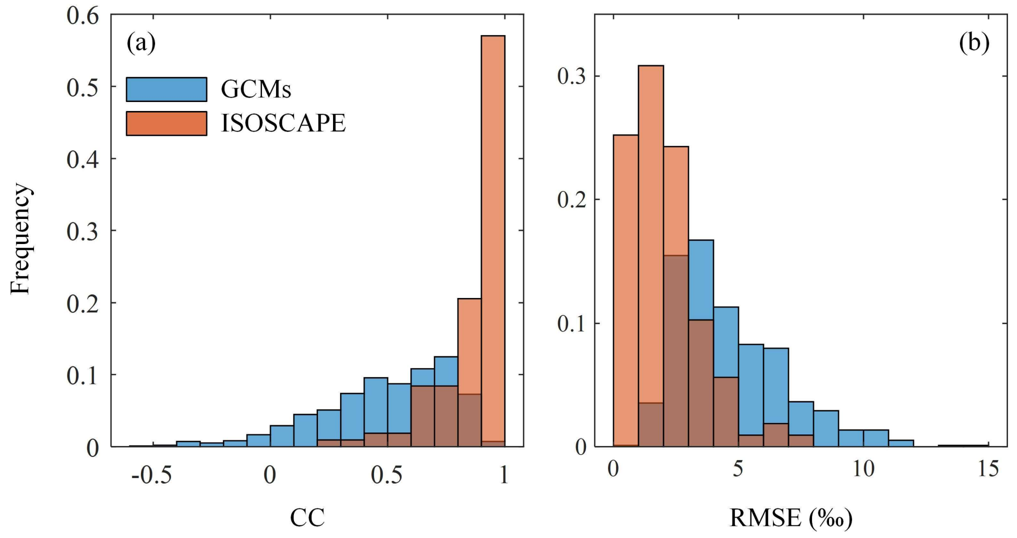

Figure S1. Histograms of correlation coefficients (CCs) and root mean square errors (RMSEs) between $\delta^{18}O_p$ observations and raw iGCM simulations and between observations and the fused isoscape for all stations.

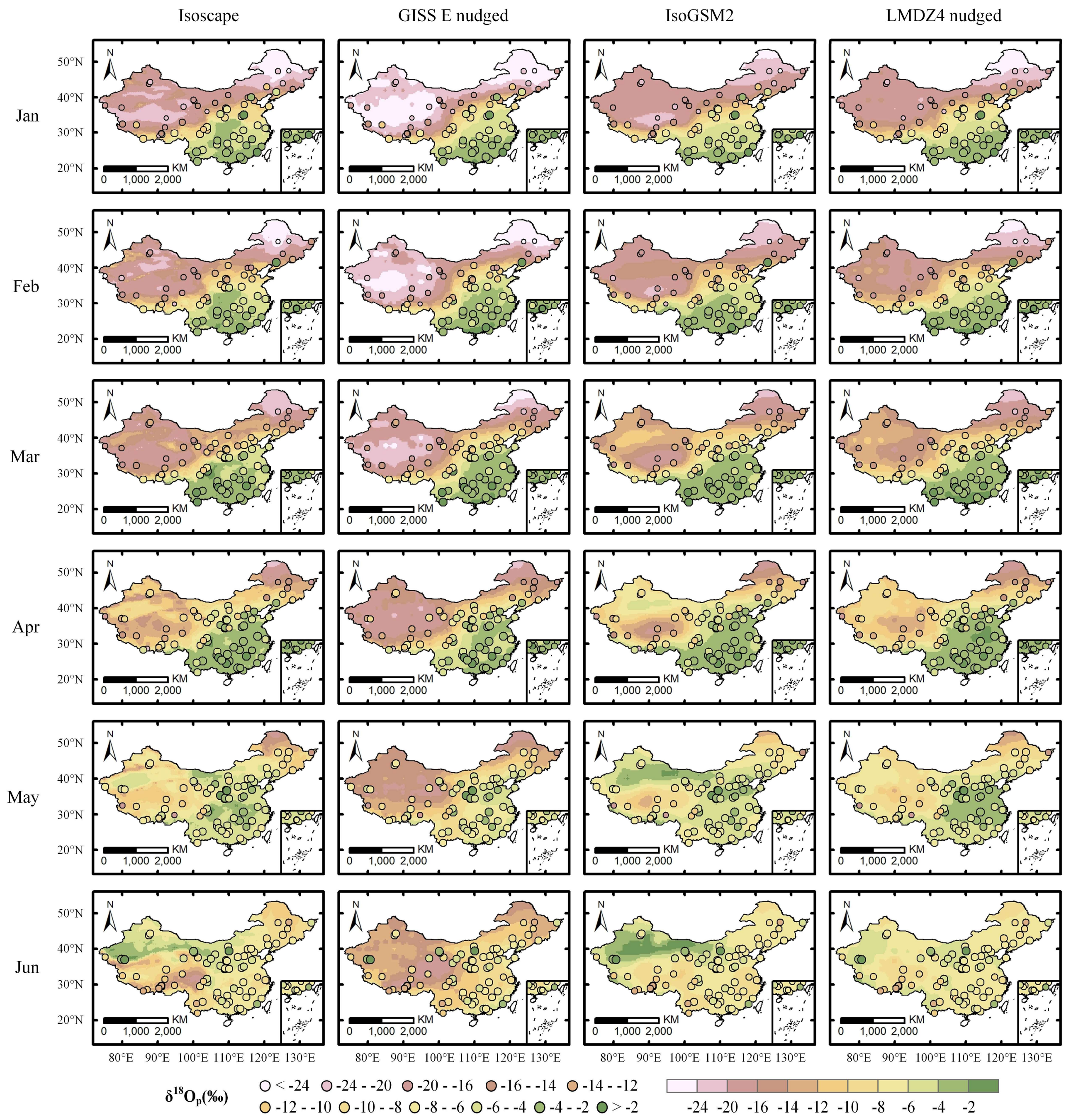

Figure S2A. Spatial distributions of $\delta^{18}O_p$ in each month for the period of 1979-2007 as obtained from observations (circles), the built isoscape (left column), and better-performing iGCMs (right three columns).

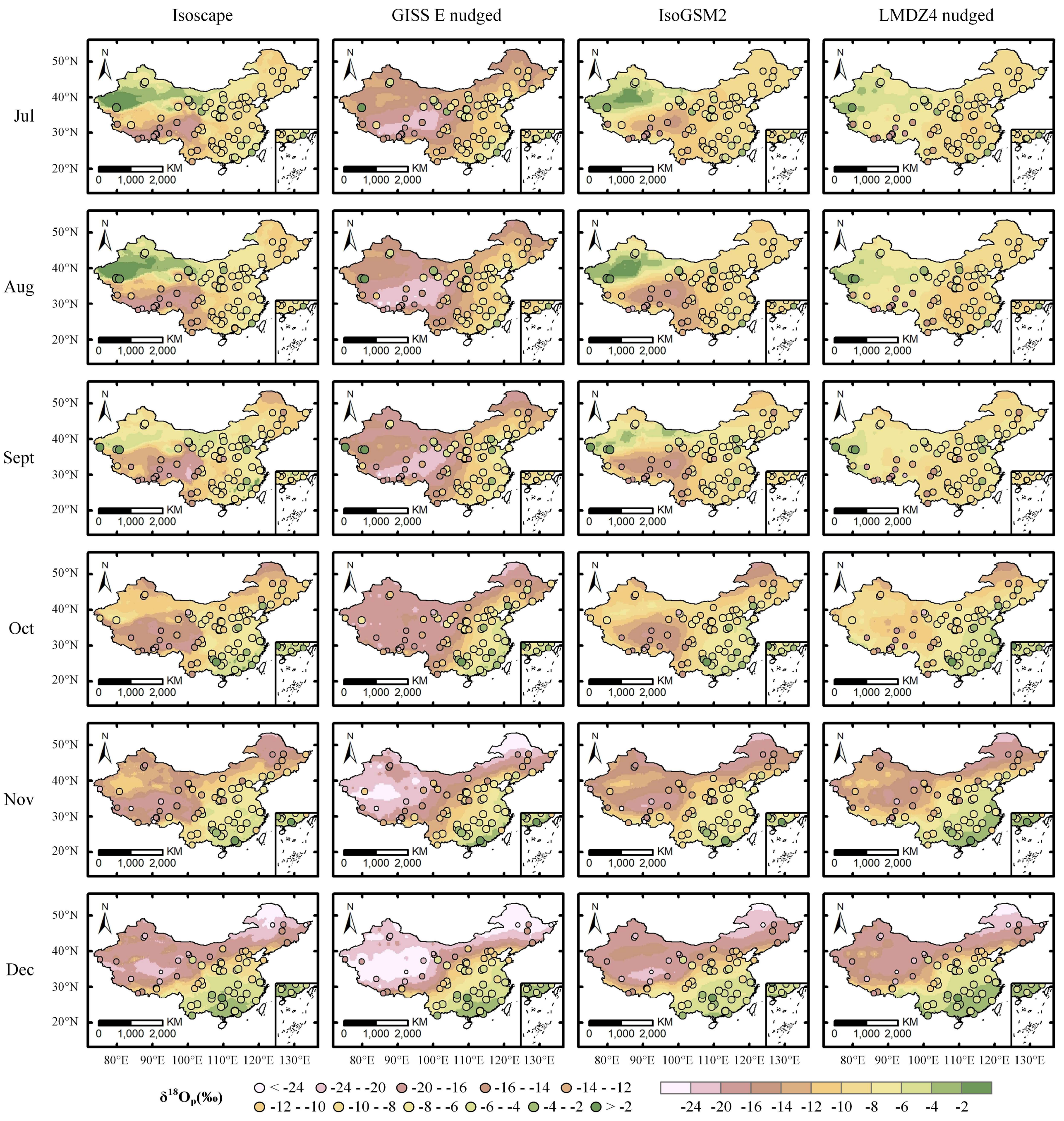

Figure S2B. Spatial distributions of $\delta^{18}O_p$ in each month for the period of 1979-2007 as obtained from observations (circles), the built isoscape (left column), and better-performing iGCMs (right three columns).

Supplementary Tables

Table S1. Detailed information and sources for each observation site.

Sub-region	Site	Latitude	Longitude	Altitude (m)	Period	Data volume	Data source
NE	Changchun	43.90	125.22	237	1999-2001	22	GNIP[1]
NE	Haerbin	45.68	126.62	172	1986-1997	35	GNIP[1]
NE	Qiqihar	47.38	123.92	147	1988-1992	50	GNIP[1]
NE	Changbaishan	42.40	128.11	738.1	2005-2010	72	CHNIP[2]
NE	Hailun	47.45	126.93	236	2005-2009	24	CHNIP[2]
NE	Sanjiang	47.35	133.30	55	2005-2007	32	CHNIP[2]
NE	Shenyang	41.52	123.37	49	2005-2010	33	CHNIP[2]
NC	Jinzhou	41.13	121.10	66	1987-1989	12	GNIP[1]
NC	Shijiazhuang	38.03	114.42	80	1985-2003	146	GNIP[1]
NC	Taiyuan	37.78	112.55	778	1986-1988	20	GNIP[1]
NC	Tianjin	39.10	117.17	3	1988-2001	64	GNIP[1]
NC	Xian	34.30	108.93	397	1985-1992	60	GNIP[1]
NC	Yantai	37.53	121.40	47	1986-1991	44	GNIP[1]
NC	Zhengzhou	34.72	113.65	110	1985-1992	57	GNIP[1]
NC	Beijing	39.96	115.43	1248	2005-2010	44	CHNIP[2]
NC	Changwu	35.24	107.68	1200	2005-2009	22	CHNIP[2]
NC	Fengqiu	35.01	114.33	67.5	2005-2007	27	CHNIP[2]
NC	Yucheng	36.83	116.57	22	2005-2008	32	CHNIP[2]
NC	Shihua	39.78	115.93	251	2011-2014	24	Duan, et al. [3]
NC	Beijing	40.00	116.39	45	2006-2015	68	Tang, et al. [4]
NC	Gaoling	34.53	109.08	381	1986-1987	11	Zhang [5]
NC	Huayin	34.47	110.08	1480	1986-1987	11	Zhang [5]
SE	Changsha	28.20	113.07	37	1988-1992	57	GNIP[1]
SE	Fuzhou	26.08	119.28	16	1985-1992	71	GNIP[1]
SE	Guangzhou	23.13	113.32	7	1986-1989	30	GNIP[1]
SE	Guilin	25.07	110.08	170	1983-1990	92	GNIP[1]
SE	Hong Kong	22.32	114.17	66	1961-2018	549	GNIP[1]
SE	Liuzhou	24.35	109.40	97	1988-1992	53	GNIP[1]
SE	Nanjing	32.18	118.18	26	1987-1992	58	GNIP[1]
SE	Wuhan	30.62	114.13	23	1986-1998	50	GNIP[1]
SE	Changshu	31.33	120.42	3.1	2005-2006	21	CHNIP[2]
SE	Dinghushan	23.16	112.55	90	2005-2010	24	CHNIP[2]
SE	Qianyanzhou	26.44	115.03	76.4	2005-2007	33	CHNIP[2]
SE	Taoyuan	28.93	111.44	106	2005-2010	47	CHNIP[2]
SE	Yingtan	28.12	116.56	45	2005-2010	56	CHNIP[2]
SE	Xiamen	24.44	118.09	24	2004-2006	18	Chen, et al. [6]
SE	Baojinggong	24.12	113.35	610	2011-2014	25	Duan, et al. [3]
SE	Penglaixian	30.23	117.53	170	2011-2014	28	Duan, et al. [3]
SE	Yongan	28.89	120.85	23	2014-2018	34	Hu, et al. [7,8]

Sub-region	Site	Latitude	Longitude	Altitude (m)	Period	Data volume	Data source
SE	Nanjing	32.12	118.95	55	2011-2018	82	Li, et al. [9]
SE	Guangzhou	23.15	113.35	39	2007-2009	34	Xie, et al. [10]
SE	Guangzhou	23.13	113.32	7	2007-2014	94	Yang, et al. [11,12]
SE	Changsha	28.25	112.55	37	2010-2017	95	Zhou, et al. [13,14]
SW	Chengdu	30.67	104.02	506	1986-1998	67	GNIP[1]
SW	Guiyang	26.58	106.72	1071	1988-1992	58	GNIP[1]
SW	Kunming	25.02	102.68	1892	1986-2003	152	GNIP[1]
SW	Zunyi	27.70	106.88	844	1986-1992	74	GNIP[1]
SW	Ailaoshan	24.55	101.03	2481	2005-2007	28	CHNIP[2]
SW	Huitong	26.85	109.61	541	2005-2010	48	CHNIP[2]
SW	Yanting	31.27	105.46	420	2009-2010	18	CHNIP[2]
SW	Yangkou	29.03	107.18	2140	2012-2016	57	Chen and Li [15]
SW	Furong	29.22	107.90	480	2011-2014	31	Duan, et al. [3]
SW	Liangfeng	26.27	108.05	600	2011-2014	29	Duan, et al. [3]
SW	Wanxiang	33.32	105.00	1200	2011-2014	25	Duan, et al. [3]
SW	Xianren	24.12	104.13	1443	2011-2013	18	Duan, et al. [3]
SW	Xishuangbanna	21.93	101.27	750	2002-2004	33	Liu, et al. [16]
SW	Duyun	25.98	107.27	1020	2003-2004	14	Luo, et al. [17]
SW	Libo	25.27	108.05	620	2003-2004	15	Luo, et al. [17]
SW	Luanchuan	33.77	111.57	900	2010-2016	79	Sun, et al. [18]
SW	Baokang	31.58	111.23	800	2013-2016	33	Wang, et al. [19]
SW	Heshang	30.45	110.42	270	2011-2018	80	Wang, et al. [20]
SW	Chongqing	29.82	106.42	252	2010-2015	65	Zhou and Li [21]
TP	Lhasa	29.70	91.13	3649	1986-1992	42	GNIP[1]
TP	Gonggashan	29.58	102.00	2950	2005-2010	32	CHNIP[2]
TP	Haibei	37.56	101.31	3280	2005-2009	23	CHNIP[2]
TP	Lhasa	29.41	91.21	3688	2005-2009	22	CHNIP[2]
TP	Maoxian	31.70	103.90	1826	2005-2009	52	CHNIP[2]
TP	Baidi	29.12	90.43	4430	2004-2007	115	TNIP[22,23]
TP	Delingha	37.37	97.37	2981	1992-2006	59	TNIP[22,23]
TP	Dingri	28.65	87.12	4330	2000-2006	37	TNIP[22,23]
TP	Dui	28.58	90.53	5030	2004-2007	45	TNIP[22,23]
TP	Gaize	32.30	84.07	4430	1998-2005	24	TNIP[22,23]
TP	Lhasa	29.70	91.13	3658	1994-2006	85	TNIP[22,23]
TP	Nagqu	31.48	92.07	4508	1999-2005	21	TNIP[22,23]
TP	Nyalam	28.18	85.97	3810	1996-2006	78	TNIP[22,23]
TP	Shiquanhe	32.50	80.08	4278	1999-2002	104	TNIP[22,23]
TP	Taxkorgen	37.77	75.27	3100	2003-2005	22	TNIP[22,23]
TP	Tuotuohe	34.22	92.43	4533	1991-2005	24	TNIP[22,23]
TP	Wengguo	28.90	90.35	4500	2004-2007	22	TNIP[22,23]
TP	Yushu	33.02	97.02	3682	2000-2004	14	TNIP[22,23]
TP	Xihexiu	36.98	76.67	2960	2012-2013	14	Sun, et al. [24]
TP	Wolong	30.86	102.97	2805	2003-2005	24	Xu, et al. [25]

Sub-region	Site	Latitude	Longitude	Altitude (m)	Period	Data volume	Data source
TP	Lulang	29.77	94.73	3330	2007-2014	79	Yang, et al. [11,12]
TP	Nuxia	29.47	94.65	2920	2009-2014	43	Yang, et al. [11,12]
TP	Yeniugou	38.46	99.54	3320	2008-2009	13	Zhao, et al. [26]
TP	Lenglong	37.55	101.83	3553	2016-2017	13	Zhu, et al. [27]
TP	Ningchan	37.68	101.88	2721	2016-2017	11	Zhu, et al. [27]
NW	Baotou	40.67	109.85	1067	1986-1992	61	GNIP[1]
NW	Hetian	37.13	79.93	1375	1988-1992	47	GNIP[1]
NW	Lanzhou	36.05	103.88	1517	1985-1999	41	GNIP[1]
NW	Wulumuqi	43.78	87.62	918	1986-2003	131	GNIP[1]
NW	Yinchuan	38.48	106.22	1112	1988-2000	30	GNIP[1]
NW	Zhangye	38.93	100.43	1483	1986-2003	86	GNIP[1]
NW	Ansai	36.86	109.32	1083	2005-2010	47	CHNIP[2]
NW	Cele	37.02	80.73	1306	2005-2007	13	CHNIP[2]
NW	Erdos	39.49	110.19	1270	2006-2009	28	CHNIP[2]
NW	Fukang	44.29	87.93	460	2005-2009	28	CHNIP[2]
NW	Linze	39.35	100.13	1375	2005-2009	46	CHNIP[2]
NW	Naiman	42.93	120.70	363	2005-2010	11	CHNIP[2]
NW	Shapotou	37.28	105.00	1350	2005-2010	28	CHNIP[2]
NW	Alxa Youqi	39.22	101.68	1620	2013-2016	30	Rao, et al. [28]
NW	Yabulai	39.30	102.70	1250	2013-2016	27	Rao, et al. [28]
NW	Jiangka	37.73	77.25	1507	2011-2013	17	Sun, et al. [24]
NW	Xinyuan	43.43	83.82	478	2015-2016	12	Wang, et al. [29]
NW	Yining	43.92	81.32	260.1	2015-2016	12	Wang, et al. [29]
NW	Zhaosu	43.16	81.13	510.9	2015-2016	12	Wang, et al. [29]
NW	Yanan	36.58	109.48	1200	1986-1987	12	Zhang [5]

Table S2. Complete hyperparameter settings of the neural network fusion methods.

Models	Hyperparameters	Values
BP	Hidden layers	3
	Dense neurons	16/32/64
	Activation function	Rectified Linear Unit (ReLU)
	Learning rate	0.005
	Batch size	20
	Loss function	Mean Square Error (MSE)
	Optimizer	Stochastic Gradient Descent (SGD)
	Epochs	1500
	Early stopping patience	150
LSTM	LSTM layers	3
	LSTM neurons	32
	Activation function	Hyperbolic Tangent (TanH)
	Dropout layers	3
	Dropout rate	0.1
	Time steps	2
	Learning rate	0.001
	Batch size	50
	Loss function	MSE
	Optimizer	Adaptive Moment Estimation (Adam)
	Epochs	1000
	Early stopping patience	100
CNN	Convolutional layers	2
	Filters	8/32
	Kernel size	4
	Activation function	ReLU
	Max pooling layers	2
	Pool size	2
	Batch normalization layers	2
	Dense layers	1
	Dense neurons	16
	Learning rate	0.0005
	Batch size	50
	Loss function	MSE
	Optimizer	Adam
	Epochs	1000
	Early stopping patience	100

Table S3. Correlation coefficient (CC) metrics of $\delta^{18}O_p$ series between observations and iGCM simulations and the built isoscape at all stations.

Subregion	Station	Isoscape	CAM	GISSf	GISSn	HadAM	IsoGSM	LMDZf	LMDZn	LMDZz	MIROC
NE	Changchun	0.988	0.798	0.744	0.773	0.642	0.802	0.676	0.863	0.634	0.685
NE	Haerbin	0.997	0.377	0.329	0.658	0.359	0.583	0.448	0.526	0.475	0.464
NE	Qiqihar	0.997	0.713	0.632	0.778	0.581	0.839	0.744	0.811	0.790	0.678
NE	Changbaishan	0.741	/	0.462	0.597	/	0.634	0.591	0.724	0.511	0.578
NE	Hailun	0.959	/	0.741	0.729	/	0.749	0.716	0.758	0.741	0.690
NE	Sanjiang	0.987	/	0.458	0.515	/	0.602	0.549	0.631	0.649	0.376
NE	Shenyang	0.645	/	0.024	0.132	/	0.314	-0.247	0.177	-0.091	0.049
NC	Jinzhou	0.728	-0.189	0.033	0.341	0.302	-0.337	0.415	0.477	-0.460	0.248
NC	Shijiazhuang	0.991	0.334	0.284	0.333	0.219	0.650	0.306	0.548	0.267	0.323
NC	Taiyuan	0.999	0.173	0.269	-0.157	-0.377	0.024	-0.116	-0.091	-0.140	0.265
NC	Tianjin	0.987	0.426	0.506	0.290	0.408	0.690	0.426	0.663	0.115	0.404
NC	Xian	0.946	0.116	-0.020	0.412	-0.229	0.658	0.201	0.594	0.298	0.411
NC	Yantai	0.972	0.177	0.075	0.457	0.234	0.485	0.162	0.480	-0.274	0.230
NC	Zhengzhou	0.992	0.072	0.182	0.487	0.000	0.630	0.215	0.559	0.083	0.390
NC	Beijing	0.835	/	0.749	0.232	/	0.702	0.779	0.739	0.596	0.801
NC	Changwu	0.881	/	-0.358	0.499	/	0.728	-0.017	0.690	0.104	0.072
NC	Fengqiu	0.990	/	0.049	0.774	/	0.704	0.261	0.664	0.095	0.201
NC	Yucheng	0.878	/	0.284	0.299	/	0.567	0.249	0.541	0.085	0.470
NC	Shihua	0.261	/	/	/	/	0.500	/	/	-0.094	/
NC	Beijing	0.736	/	0.520	0.468	/	0.579	0.277	0.579	0.322	0.556
NC	Gaoling	0.921	0.005	0.153	0.390	-0.177	0.382	0.172	0.465	0.649	0.291
NC	Huayin	0.934	0.038	-0.110	0.609	-0.286	0.325	-0.087	0.617	0.799	0.461
SE	Changsha	0.992	0.449	0.504	0.693	0.158	0.821	0.563	0.756	0.625	0.531
SE	Fuzhou	0.888	0.237	0.091	0.500	0.237	0.587	0.229	0.372	0.522	0.333
SE	Guangzhou	0.991	0.365	0.601	0.600	0.466	0.611	0.608	0.582	0.443	0.439
SE	Guilin	0.991	0.594	0.546	0.863	0.428	0.877	0.713	0.785	0.788	0.708
SE	Hong Kong	0.903	0.606	0.604	0.791	0.644	0.796	0.681	0.777	0.642	0.616
SE	Liuzhou	0.978	0.527	0.517	0.744	0.489	0.740	0.636	0.740	0.717	0.708
SE	Nanjing	0.988	0.252	0.366	0.621	0.308	0.764	0.340	0.727	0.345	0.241
SE	Wuhan	0.994	0.367	0.253	0.637	0.088	0.713	0.356	0.697	0.640	0.505
SE	Changshu	0.986	/	0.288	0.789	/	0.719	0.408	0.736	0.733	0.133
SE	Dinghushan	0.522	/	0.394	0.686	/	0.463	0.968	0.999	0.492	0.997
SE	Qianyanzhou	0.821	/	0.123	0.591	/	0.725	0.401	0.631	0.590	0.543
SE	Taoyuan	0.912	/	0.532	0.821	/	0.819	0.556	0.762	0.782	0.401
SE	Yingtan	0.800	/	0.071	0.346	/	0.443	0.307	0.225	0.419	0.180
SE	Xiamen	0.843	/	0.514	0.506	/	0.504	0.511	0.320	0.390	0.170
SE	Baojinggong	0.584	/	/	/	/	0.600	/	/	0.496	/
SE	Penglaixian	0.486	/	/	/	/	0.547	/	/	0.401	/
SE	Yongan	0.376	/	/	/	/	0.337	/	/	0.353	/
SE	Nanjing	0.652	/	/	/	/	0.727	/	/	0.447	/
SE	Guangzhou	0.867	/	0.510	0.826	/	0.717	0.794	0.760	0.734	0.641

Subregion	Station	Isoscape	CAM	GISSf	GISSn	HadAM	IsoGSM	LMDZf	LMDZn	LMDZz	MIROC
SE	Guangzhou	0.851	/	0.468	0.838	/	0.830	0.914	0.834	0.636	0.765
SE	Changsha	0.865	/	/	/	/	0.874	/	/	0.795	/
SW	Chengdu	0.998	0.432	0.167	0.671	-0.055	0.703	0.387	0.684	0.663	0.523
SW	Guiyang	0.994	0.306	0.458	0.745	0.249	0.848	0.545	0.779	0.815	0.552
SW	Kunming	0.996	0.554	0.617	0.817	0.469	0.876	0.650	0.860	0.662	0.680
SW	Zunyi	0.956	0.294	0.411	0.759	0.236	0.843	0.538	0.802	0.787	0.507
SW	Ailaoshan	0.928	/	0.659	0.872	/	0.791	0.705	0.828	0.143	0.795
SW	Huitong	0.917	/	0.447	0.773	/	0.820	0.692	0.802	0.779	0.450
SW	Yanting	0.753	/	0.316	0.428	/	0.734	/	/	0.716	/
SW	Yangkou	0.801	/	/	/	/	0.784	/	/	0.860	/
SW	Furong	0.802	/	/	/	/	0.722	/	/	0.730	/
SW	Liangfeng	0.495	/	/	/	/	0.435	/	/	0.429	/
SW	Wanxiang	0.801	/	/	/	/	0.725	/	/	0.783	/
SW	Xianren	0.876	/	/	/	/	0.810	/	/	0.799	/
SW	Xishuangbanna	0.984	0.532	0.819	0.850	/	0.767	0.567	0.780	0.680	0.784
SW	Duyun	0.990	0.247	0.571	0.739	/	0.829	0.745	0.763	0.862	0.724
SW	Libo	0.975	0.612	0.760	0.770	/	0.875	0.753	0.860	0.760	0.586
SW	Luanchuan	0.633	/	/	/	/	0.629	/	/	0.395	/
SW	Baokang	0.785	/	/	/	/	0.739	/	/	0.658	/
SW	Heshang	0.863	/	/	/	/	0.866	/	/	0.734	/
SW	Chongqing	0.841	/	/	/	/	0.852	/	/	0.784	/
TP	Lhasa	0.977	0.192	0.393	0.593	0.371	0.538	0.132	0.128	0.190	0.201
TP	Gonggashan	0.685	/	0.098	0.681	/	0.806	0.319	0.617	0.693	0.358
TP	Haibei	0.963	/	0.242	0.303	/	0.488	0.643	0.502	0.433	0.401
TP	Lhasa	0.718	/	0.578	0.590	/	0.521	0.362	0.186	0.275	0.451
TP	Maoxian	0.623	/	-0.392	0.477	/	0.772	0.069	0.657	0.450	0.127
TP	Baidi	0.918	/	0.511	0.617	/	0.456	0.293	0.469	0.444	0.494
TP	Delingha	0.997	0.272	0.451	0.753	0.749	0.736	0.804	0.855	0.803	0.734
TP	Dingri	0.935	-0.077	0.613	0.178	0.712	0.300	-0.325	0.438	0.373	0.348
TP	Dui	0.911	/	0.564	0.534	/	0.488	0.337	0.281	0.385	0.429
TP	Gaize	0.993	0.342	0.032	0.441	-0.059	0.576	0.356	0.369	0.433	0.179
TP	Lhasa	0.948	0.365	0.627	0.744	0.446	0.649	0.451	0.396	0.417	0.556
TP	Nagqu	0.982	0.172	0.165	0.641	0.154	0.377	0.150	0.058	0.458	0.403
TP	Nyalam	0.948	0.403	0.456	0.689	-0.060	0.650	0.478	0.319	0.351	0.484
TP	Shiquanhe	0.984	-0.331	0.254	0.350	-0.056	0.448	0.457	0.350	0.426	0.045
TP	Taxkorgen	0.966	0.186	0.699	0.790	/	0.810	0.800	0.817	0.832	0.698
TP	Tuotuohe	0.993	0.178	0.099	0.644	0.436	0.791	0.665	0.667	0.668	0.476
TP	Wengguo	0.898	/	0.656	0.573	/	0.712	0.291	0.530	0.626	0.562
TP	Yushu	0.908	0.004	-0.124	0.370	-0.111	0.469	0.180	0.279	0.461	0.293
TP	Xihexiu	0.861	/	/	/	/	0.836	/	/	0.945	/
TP	Wolong	0.677	-0.424	0.091	0.297	/	0.588	0.051	0.603	0.381	0.198
TP	Lulang	0.868	/	0.452	0.761	/	0.773	0.635	0.618	0.679	0.272
TP	Nuxia	0.665	/	-0.212	0.793	/	0.671	/	/	0.494	/

Subregion	Station	Isoscape	CAM	GISSf	GISSn	HadAM	IsoGSM	LMDZf	LMDZn	LMDZz	MIROC
TP	Yeniugou	0.964	/	0.876	0.835	/	0.863	/	/	0.880	/
TP	Lenglong	0.911	/	/	/	/	0.595	/	/	0.856	/
TP	Ningchan	0.619	/	/	/	/	0.041	/	/	0.746	/
NW	Baotou	0.980	0.432	0.517	0.510	0.393	0.613	0.431	0.600	0.282	0.459
NW	Hetian	0.975	0.487	0.519	0.822	0.547	0.872	0.812	0.892	0.880	0.823
NW	Lanzhou	0.910	0.183	0.220	-0.070	0.526	0.387	0.601	0.605	0.451	0.607
NW	Wulumuqi	0.981	0.396	0.672	0.764	0.759	0.818	0.782	0.862	0.869	0.730
NW	Yinchuan	0.976	0.433	0.582	0.338	0.453	0.668	0.623	0.763	0.753	0.634
NW	Zhangye	0.966	0.350	0.586	0.565	0.625	0.683	0.797	0.829	0.795	0.755
NW	Ansai	0.880	/	-0.065	0.525	/	0.682	-0.036	0.663	0.003	0.330
NW	Cele	0.985	/	-0.381	0.656	/	0.700	0.592	0.787	0.719	0.555
NW	Erdos	0.752	/	0.006	0.703	/	0.797	0.331	0.131	0.171	-0.542
NW	Fukang	0.967	/	0.752	0.778	/	0.881	0.873	0.884	0.859	0.838
NW	Linze	0.972	/	0.676	0.674	/	0.818	0.779	0.786	0.681	0.806
NW	Naiman	0.926	/	0.622	0.636	/	0.651	0.554	0.417	0.745	0.106
NW	Shapotou	0.671	/	0.140	0.202	/	0.309	0.300	0.303	-0.096	0.165
NW	Alxa Youqi	0.917	/	/	/	/	0.807	/	/	0.874	/
NW	Yabulai	0.819	/	/	/	/	0.686	/	/	0.742	/
NW	Jiangka	0.946	/	/	/	/	0.940	/	/	0.943	/
NW	Xinyuan	0.730	/	/	/	/	0.294	/	/	0.874	/
NW	Yining	0.794	/	/	/	/	0.696	/	/	0.657	/
NW	Zhaosu	0.881	/	/	/	/	0.724	/	/	0.889	/
NW	Yanan	0.968	0.326	0.354	0.162	0.146	0.623	0.256	0.554	0.407	0.179

Table S4. Root mean square error (RMSE, ‰) metrics of $\delta^{18}O_p$ series between observations and iGCM simulations and the built isoscape at all stations.

Subregion	Station	Isoscape	CAM	GISSf	GISSn	HadAM	IsoGSM	LMDZf	LMDZn	LMDZz	MIROC
NE	Changchun	0.636	2.451	3.137	3.194	3.699	3.007	3.442	2.440	4.725	2.942
NE	Haerbin	0.286	3.513	4.150	5.245	3.753	2.604	3.442	2.759	4.048	3.496
NE	Qiqihar	0.641	5.401	6.096	4.892	7.119	4.613	5.558	4.765	5.428	5.454
NE	Changbaishan	3.435	/	6.131	6.038	/	3.881	3.791	3.077	5.387	3.654
NE	Hailun	2.087	/	4.688	5.731	/	5.492	5.246	5.144	4.697	5.058
NE	Sanjiang	1.208	/	6.966	6.894	/	5.138	6.487	5.099	5.023	6.058
NE	Shenyang	2.824	/	4.826	3.941	/	3.732	6.384	3.656	5.787	5.021
NC	Jinzhou	1.685	3.142	2.382	1.903	2.300	3.234	1.894	1.928	6.212	3.263
NC	Shijiazhuang	0.586	4.659	4.088	3.780	3.586	2.902	3.678	2.994	6.948	4.341
NC	Taiyuan	0.218	5.130	5.239	5.492	4.681	4.480	4.322	4.646	7.549	4.268
NC	Tianjin	0.573	3.889	3.189	3.113	3.224	3.103	3.079	2.347	6.830	3.919
NC	Xian	1.106	5.292	4.573	4.243	4.147	2.437	3.660	2.700	3.933	3.435
NC	Yantai	0.630	2.896	3.230	2.742	2.828	2.739	3.140	2.598	5.572	3.751
NC	Zhengzhou	0.473	4.796	4.254	3.124	3.550	2.571	3.327	2.719	5.568	3.107
NC	Beijing	3.504	/	3.429	4.577	/	3.908	3.114	3.024	4.393	2.283
NC	Changwu	2.419	/	7.183	6.633	/	3.979	4.172	2.634	5.484	5.447
NC	Fengqiu	0.732	/	4.849	3.668	/	3.022	3.889	3.124	5.945	4.145
NC	Yucheng	1.808	/	4.061	3.816	/	3.161	4.194	3.777	6.048	3.730
NC	Shihua	3.921	/	/	/	/	3.831	/	/	7.375	/
NC	Beijing	2.397	/	3.394	3.643	/	3.218	5.101	3.544	5.218	4.446
NC	Gaoling	1.386	6.937	4.898	4.569	2.939	2.907	3.129	2.454	4.359	3.030
NC	Huayin	2.376	5.128	4.456	3.254	4.397	4.090	4.935	4.096	2.283	3.761
SE	Changsha	0.410	2.892	3.177	2.924	3.622	1.856	2.602	2.065	2.564	2.612
SE	Fuzhou	1.300	3.226	3.361	2.661	3.215	2.499	3.340	3.061	2.582	3.051
SE	Guangzhou	0.353	2.760	1.964	1.992	2.997	2.408	2.016	2.393	3.142	2.419
SE	Guilin	0.437	2.585	2.574	2.022	3.033	1.570	2.243	2.005	1.925	2.252
SE	Hong Kong	1.165	2.264	2.405	1.730	2.685	1.737	2.348	1.824	2.176	2.275
SE	Liuzhou	0.665	2.858	2.728	2.290	2.901	2.260	2.504	2.211	2.432	2.258
SE	Nanjing	0.438	2.907	2.523	2.234	3.021	1.796	3.359	2.666	2.905	3.256
SE	Wuhan	0.316	2.785	3.530	2.740	3.448	2.138	2.877	2.212	2.349	2.492
SE	Changshu	0.455	/	2.157	1.250	/	1.412	3.161	2.328	1.492	2.530
SE	Dinghushan	4.320	/	4.477	4.158	/	4.729	2.139	0.692	4.581	3.114
SE	Qianyanzhou	1.483	/	3.631	3.149	/	2.416	2.502	2.334	2.425	2.320
SE	Taoyuan	1.228	/	2.773	2.397	/	1.515	2.475	2.059	2.075	2.943
SE	Yingtan	1.596	/	3.219	3.560	/	2.983	2.317	3.170	2.947	2.428
SE	Xiamen	1.453	/	1.538	1.987	/	2.859	1.597	3.075	2.536	2.320
SE	Baojinggong	2.615	/	/	/	/	2.419	/	/	2.990	/
SE	Penglaixian	2.962	/	/	/	/	2.745	/	/	3.265	/
SE	Yongan	2.693	/	/	/	/	2.869	/	/	2.858	/
SE	Nanjing	2.086	/	/	/	/	1.891	/	/	2.827	/
SE	Guangzhou	1.584	/	2.908	1.916	/	2.050	2.717	2.645	2.069	2.842

Subregion	Station	Isoscape	CAM	GISSf	GISSn	HadAM	IsoGSM	LMDZf	LMDZn	LMDZz	MIROC
SE	Guangzhou	1.549	/	2.799	1.646	/	1.743	1.647	1.789	2.426	1.989
SE	Changsha	1.720	/	/	/	/	1.423	/	/	2.218	/
SW	Chengdu	0.387	6.873	6.962	7.582	4.807	3.894	3.612	3.147	3.202	5.319
SW	Guiyang	0.423	4.195	3.529	3.057	4.058	1.988	3.385	2.525	2.920	3.289
SW	Kunming	0.448	3.872	3.609	3.616	3.940	2.121	3.866	3.318	3.867	3.652
SW	Zunyi	1.144	4.472	3.889	3.609	4.007	2.057	3.308	2.448	2.574	3.357
SW	Ailaoshan	2.041	/	3.820	3.008	/	3.014	5.154	4.671	6.331	4.901
SW	Huitong	1.196	/	3.072	2.567	/	1.684	1.921	1.699	2.108	2.802
SW	Yanting	5.417	/	10.513	11.162	/	6.017	/	/	4.735	/
SW	Yangkou	2.984	/	/	/	/	2.799	/	/	3.302	/
SW	Furong	2.156	/	/	/	/	2.508	/	/	2.362	/
SW	Liangfeng	4.259	/	/	/	/	4.382	/	/	4.586	/
SW	Wanxiang	1.815	/	/	/	/	2.310	/	/	2.000	/
SW	Xianren	1.926	/	/	/	/	2.329	/	/	3.070	/
SW	Xishuangbanna	0.998	4.314	3.403	2.881	/	3.583	5.634	5.123	4.893	5.607
SW	Duyun	0.746	4.313	3.476	3.284	/	2.319	2.915	2.546	2.741	2.887
SW	Libo	0.762	4.132	2.975	3.287	/	2.224	1.997	1.938	2.241	2.563
SW	Luanchuan	2.417	/	/	/	/	2.346	/	/	3.309	/
SW	Baokang	2.204	/	/	/	/	2.202	/	/	2.575	/
SW	Heshang	1.882	/	/	/	/	1.880	/	/	2.521	/
SW	Chongqing	2.318	/	/	/	/	2.523	/	/	2.749	/
TP	Lhasa	1.808	7.004	7.443	6.259	6.516	6.592	7.762	8.234	6.764	7.129
TP	Gonggashan	3.385	/	4.230	5.023	/	3.023	4.682	4.280	4.193	4.237
TP	Haibei	1.341	/	8.004	9.464	/	4.893	4.721	4.463	7.080	6.703
TP	Lhasa	4.750	/	5.411	5.393	/	7.175	7.478	8.149	6.501	6.568
TP	Maoxian	2.952	/	5.509	6.292	/	2.266	3.346	2.374	3.341	3.221
TP	Baidi	1.996	/	4.619	4.348	/	6.210	7.136	6.999	5.037	7.729
TP	Delingha	0.756	9.141	11.921	10.557	6.185	4.871	3.816	3.336	7.992	6.659
TP	Dingri	1.953	6.037	3.760	5.519	6.769	8.006	8.995	8.814	4.384	8.598
TP	Dui	2.995	/	5.869	5.716	/	8.075	8.785	8.924	6.952	9.136
TP	Gaize	0.951	7.348	10.741	8.043	6.897	5.411	7.624	7.515	6.010	7.021
TP	Lhasa	2.340	6.118	5.548	5.111	6.438	6.027	8.229	8.586	6.229	6.628
TP	Nagqu	1.191	6.421	5.585	4.580	5.689	5.343	8.058	8.112	5.277	4.590
TP	Nyalam	2.018	5.765	5.460	4.231	9.508	6.422	6.349	6.569	5.948	7.325
TP	Shiquanhe	1.844	7.724	6.783	6.701	6.888	9.335	10.376	10.032	7.331	10.522
TP	Taxkorgen	3.552	6.634	9.574	10.306	/	6.188	6.763	6.474	6.960	7.427
TP	Tuotuohe	0.830	8.888	11.023	7.661	5.356	3.914	5.523	5.470	4.451	5.904
TP	Wengguo	2.391	/	4.108	4.699	/	6.935	7.930	7.926	5.256	8.400
TP	Yushu	1.359	6.320	8.266	6.229	3.496	3.819	5.242	4.852	3.402	4.838
TP	Xihexiu	4.580	/	/	/	/	5.320	/	/	3.089	/
TP	Wolong	2.242	3.558	2.273	3.443	/	4.238	4.839	4.542	4.773	2.971
TP	Lulang	3.215	/	5.301	4.495	/	4.157	8.270	8.049	5.009	6.793
TP	Nuxia	3.118	/	4.609	6.384	/	3.204	/	/	3.695	/

Subregion	Station	Isoscape	CAM	GISSf	GISSn	HadAM	IsoGSM	LMDZf	LMDZn	LMDZz	MIROC
TP	Yeniugou	3.221	/	8.490	10.379	/	4.275	/	/	4.522	/
TP	Lenglong	3.060	/	/	/	/	3.984	/	/	3.008	/
TP	Ningchan	4.883	/	/	/	/	6.345	/	/	3.958	/
NW	Baotou	0.781	6.477	5.476	4.926	5.605	3.326	4.202	3.125	7.794	5.204
NW	Hetian	2.086	8.839	9.787	11.362	11.207	4.374	5.292	4.536	8.980	5.545
NW	Lanzhou	2.123	8.665	8.279	10.171	4.488	5.138	3.801	3.736	5.976	6.701
NW	Wulumuqi	1.346	5.837	5.185	6.107	6.083	4.112	4.843	3.893	6.065	4.344
NW	Yinchuan	1.106	6.992	6.082	6.544	4.971	3.622	3.838	3.353	6.126	5.457
NW	Zhangye	1.946	9.135	9.859	10.121	6.465	5.109	4.339	4.019	7.025	6.934
NW	Ansai	1.794	/	6.254	5.194	/	3.205	4.413	2.542	5.496	4.349
NW	Cele	1.263	/	13.796	14.641	/	5.253	5.115	4.896	9.039	7.019
NW	Erdos	2.667	/	6.831	6.224	/	3.511	3.139	3.819	7.335	4.863
NW	Fukang	1.831	/	5.257	6.830	/	3.612	3.944	3.610	5.970	3.951
NW	Linze	2.048	/	9.308	9.952	/	4.999	4.841	4.830	8.911	6.775
NW	Naiman	1.870	/	3.023	3.000	/	3.993	3.153	3.105	6.814	3.622
NW	Shapotou	3.023	/	8.239	10.470	/	4.910	3.503	3.947	7.864	5.781
NW	Alxa Youqi	2.776	/	/	/	/	4.082	/	/	7.415	/
NW	Yabulai	4.552	/	/	/	/	4.894	/	/	10.476	/
NW	Jiangka	3.890	/	/	/	/	5.364	/	/	5.925	/
NW	Xinyuan	6.763	/	/	/	/	10.006	/	/	7.401	/
NW	Yining	7.358	/	/	/	/	7.875	/	/	9.197	/
NW	Zhaosu	6.881	/	/	/	/	8.230	/	/	7.891	/
NW	Yanan	0.839	6.657	4.580	4.033	3.565	2.260	3.335	2.607	6.234	4.825

References

1 IAEA/WMO. Global network of isotopes in precipitation. *The GNIP Database* https://nucleus.iaea.org/wiser (2022).

2 Liu, J., Song, X., Yuan, G., Sun, X. & Yang, L. Stable isotopic compositions of precipitation in China. *Tellus B Chem. Phys. Meteorol.* **66**, 22567 (2014).

3 Duan, W. *et al.* The transfer of seasonal isotopic variability between precipitation and drip water at eight caves in the monsoon regions of China. *Geochim. Cosmochim. Acta* **183**, 250-266 (2016).

4 Tang, Y. *et al.* Using stable isotopes to understand seasonal and interannual dynamics in moisture sources and atmospheric circulation in precipitation. *Hydrol. Process.* **31**, 4682-4692 (2017).

5 Zhang, S. Characteristics of hydrogen-oxygen isotope compositions of contemporarily atmospheric sedimentation in Shaanxi Province. *Geology of Shaanxi* **7**, 57-66 (1989).

6 Chen, J., Cao, J. & Huang, Y. The hydrogen and oxygen isotope composition of precipitation in the Xiamen coastal area. *J. Mar. Sci.* **28**, 11-17 (2010).

7 Hu, M. *et al.* Assessment of streamflow components and hydrologic transit times using stable isotopes of oxygen and hydrogen in waters of a subtropical watershed in eastern China. *J. Hydrol.* **589**, 125363 (2020).

8 Chen, D. Data for: Using stable isotopes of water to estimate the young water fraction and mean transit time in a subtropical watershed of eastern China. *Mendeley Data* https://doi.org/10.17632/vndfs3dpyn.1 (2020).

9 Li, Y. *et al.* Variations of stable isotopic composition in atmospheric water vapor and their controlling factors—A 6-year continuous sampling study in Nanjing, Eastern China. *J. Geophys. Res. Atmos.* **125**, e2019JD031697 (2020).

10 Xie, L., Wei, G., Deng, W. & Zhao, X. Daily δ18O and δD of precipitations from 2007 to 2009 in Guangzhou, South China: Implications for changes of moisture sources. *J. Hydrol.* **400**, 477-489 (2011).

11 Yang, X., Davis, M. E., Acharya, S. & Yao, T. Asian monsoon variations revealed from stable isotopes in precipitation. *Clim. Dyn.* **51**, 2267-2283 (2018).

12 Yang, X. The stable oxygen isotope data of daily precipitation in Lulang, Nuxia, and Guangzhou (2007-2014). *National Tibetan Plateau/Third Pole Environment Data Center* https://doi.org/10.11888/AtmosPhys.tpe.249477.db (2018).

13 Zhou, H. *et al.* Variation of δ18O in precipitation and its response to upstream atmospheric convection and rainout: A case study of Changsha station, south-central China. *Sci. Total Environ.* **659**, 1199-1208 (2019).

14 Zhang, X. Data for: Variation of δ18O in precipitation and its response to upstream atmospheric convection and rainout: a case study of Changsha station, south-central China. *Mendeley Data* https://doi.org/10.17632/975k2wzw3p.1 (2019).

15 Chen, C.-J. & Li, T.-Y. Geochemical characteristics of cave drip water respond to ENSO based on a 6-year monitoring work in Yangkou Cave, Southwest China. *J. Hydrol.* **561**, 896-907 (2018).

16 Liu, W. J. *et al.* Using stable isotopes to determine sources of fog drip in a tropical seasonal rain forest of Xishuangbanna, SW China. *Agric. For. Meteorol.* **143**, 80-91 (2007).

17 Luo, W., Wang, S. & Xie, X. A comparative study on the stable isotopes from precipitation to speleothem in four caves of Guizhou, China. *Geochemistry* **73**, 205-215 (2013).

18 Sun, Z., Yang, Y., Zhao, J., Tian, N. & Feng, X. Potential ENSO effects on the oxygen isotope composition of modern speleothems: Observations from Jiguan Cave, central China. *J. Hydrol.* **566**, 164-174 (2018).

19 Wang, Q. *et al.* The transfer of oxygen isotopic signals from precipitation to drip water and modern calcite on the seasonal time scale in Yongxing Cave, central China. *Environ. Earth Sci.* **77**, 1-18 (2018).
20 Wang, Y., Hu, C., Ruan, J. & Johnson, K. R. East Asian precipitation δ18O relationship with various monsoon indices. *J. Geophys. Res. Atmos.* **125**, e2019JD032282 (2020).
21 Zhou, J.-L. & Li, T.-Y. A tentative study of the relationship between annual δ18O & δD variations of precipitation and atmospheric circulations—a case from Southwest China. *Quat. Int.* **479**, 117-127 (2018).
22 Yao, T. *et al.* A review of climatic controls on δ18O in precipitation over the Tibetan Plateau: Observations and simulations. *Rev. Geophys.* **51**, 525-548 (2013).
23 Gao, J. Data set of δ18O stable isotopes in precipitation from Tibetan Network for Isotopes(1991–2008). *National Tibetan Plateau Data Center* https://doi.org/10.11888/Geogra.tpdc.270940 (2020).
24 Sun, C., Chen, Y., Li, J., Chen, W. & Li, X. Stable isotope variations in precipitation in the northwesternmost Tibetan Plateau related to various meteorological controlling factors. *Atmos. Res.* **227**, 66-78 (2019).
25 Xu, Q., Liu, S., An, S., Jiang, C. & Liu, X. Study on hydrogen and oxygen stable isotopes in precipitation in Wolong Nature Reserve, Sichuan Province. *Forest Research* **19**, 679-686 (2006).
26 Zhao, L. *et al.* Isotopic evidence for the moisture origin and composition of surface runoff in the headwaters of the Heihe River basin. *Chin. Sci. Bull.* **56**, 406-415 (2011).
27 Zhu, G. *et al.* Contribution of recycled moisture to precipitation in the monsoon marginal zone: Estimate based on stable isotope data. *J. Hydrol.* **569**, 423-435 (2019).
28 Rao, W. *et al.* Identifying the source of atmospheric moisture over arid deserts using stable isotopes (2H and 18O) in precipitation. *Hydrol. Process.* **32**, 436-449 (2018).
29 Wang, L., Dong, Y., Han, D. & Xu, Z. Stable isotopic compositions in precipitation over wet island in Central Asia. *J. Hydrol.* **573**, 581-591 (2019).
